# Regulation of Cues vs Cognitive Behavioral Therapy for Binge Eating and Weight Loss Among Veterans: A Feasibility and Randomized Clinical Trial

**DOI:** 10.1001/jamanetworkopen.2025.25064

**Published:** 2025-08-04

**Authors:** Kerri N. Boutelle, Niloofar Afari, Saori Obayashi, Dawn M. Eichen, David R. Strong, Ellen K. Pasquale, Carol B. Peterson

**Affiliations:** 1 Department of Pediatrics, University of California, San Diego, La Jolla; 2 Herbert Wertheim School of Public Health and Human Longevity Science, University of California, San Diego, La Jolla; 3 Department of Psychiatry, University of California San Diego, La Jolla; 4 VA San Diego Healthcare System, San Diego, California; 5 San Diego State University/University of California, San Diego Joint Doctoral Program in Clinical Psychology, San Diego; 6 Department of Psychiatry and Behavioral Sciences, University of Minnesota, Minneapolis

## Abstract

**Importance:**

Cognitive behavioral therapy (CBT) has the most empirical support for treatment of binge eating. Appetitive traits, including food responsiveness and satiety responsiveness, impact how individuals interact with the current obesogenic environment. The regulation of cues (ROC) plus behavioral weight loss (BWL) intervention was specifically developed to target food responsiveness, satiety responsiveness, and energy reduction.

**Objective:**

To evaluate the feasibility and efficacy of ROC+BWL and CBT over 5 months of treatment and 6 months of follow-up and to explore whether clinical binge eating was a moderator of outcomes.

**Design, Setting, and Participants:**

This randomized clinical trial was conducted from March 2019 to April 2023 among veterans at a university clinic. Eligible participants were veterans who met criteria for Binge Eating Disorder (BED) or subthreshold BED, had a body mass index (BMI; calculated as weight in kilograms divided by height in meters squared) of 25 to 45, were aged 18 to 65 years, and were free of other exclusionary criteria. Data were analyzed from January 2024 to June 2025.

**Intervention:**

The ROC+BWL intervention uniquely targets food responsiveness, satiety responsiveness, and energy reduction. CBT focuses on disrupting the dietary restraint/binge eating cycle by changing maladaptive thoughts and behaviors. Participants were randomized to receive either ROC+BWL or CBT for 5 months.

**Main Outcomes and Measures:**

The main outcomes were feasibility and change in binge eating (measured as loss of control) and body weight, assessed at midtreatment (2.5 months), posttreatment (5 months), and a 6-month follow-up (11 months).

**Results:**

A total of 1853 veterans inquired about participation and 1724 were excluded or declined to participate. The final sample included 129 veterans (mean [SD] age, 47.1 [11.3] years; 76 [59%] male; mean [SD] BMI, 34.8 [4.7]), with 63 randomized to ROC+BWL and 66 to CBT. A total of 123 veterans (95%) provided data posttreatment, and 115 veterans (89%) provided data at the 6-month follow-up. Attendance and acceptability ratings did not differ between treatments. ROC+BWL resulted in a greater reduction in risk of binge eating than CBT at midtreatment (difference in probability, −0.20; 95% credible interval [CrI], −0.30 to −0.11), posttreatment (difference in probability, −0.23; 95% CrI, −0.22 to −0.19), and at the 6-month follow-up (difference in probability, −0.21; 95% CrI, −0.21 to −0.18). ROC+BWL also resulted in greater weight loss at midtreatment (difference in BMI change, −0.68; 95% CrI, −1.23 to −0.12) and posttreatment (difference in BMI change, −0.71; 95% CrI, −1.40 to −0.01) assessments than CBT, but significant differences were no longer observed at the 6-month follow-up (difference in BMI change, −0.22; 95% CrI, −0.98 to 0.54). Results were more pronounced among veterans with BED.

**Conclusions and Relevance:**

In this randomized clinical trial among veterans with binge eating and obesity, ROC+BWL resulted in greater decreases in binge eating compared with CBT. Although ROC+BWL resulted in greater weight loss compared with CBT during treatment, these differences were not maintained. Thus, ROC+BWL could be an alternate model to treat BED among veterans, but effects on weight need further research.

**Trial Registration:**

ClinicalTrials.gov Identifier: NCT03678766

## Key Points

**Question:**

Can the regulation of cues (ROC) treatment reduce binge eating and weight among veterans more than cognitive behavioral therapy (CBT)?

**Findings:**

In this randomized clinical trial with 129 veterans with binge eating and overweight or obesity, analysis showed that ROC reduced binge eating and weight more than CBT during the 5 months of treatment, and the binge eating reductions, but not weight reductions, were maintained at the 6-month follow-up. Results were more pronounced in individuals with binge eating disorder.

**Meaning:**

These finding suggest that the ROC program targeting appetitive traits could be an effective treatment to reduce binge eating among veterans, although trials with longer follow-up are needed.

## Teaser

This randomized clinical trial evaluates the feasibility and efficacy of a regulation of cues with behavioral weight loss intervention compared with cognitive behavioral therapy (CBT) to reduce body mass index and binge eating episodes among veterans with overweight or obesity and clinical or subthreshold binge eating disorder.

## Introduction

Binge eating is the consumption of a large amount of food within a discrete amount of time while experiencing loss of control (LOC) over eating.^1^ Binge eating disorder (BED), which includes recurrent binge eating episodes and marked distress, is highly comorbid with overweight and obesity.^2,3,4,5,6,7^ Cognitive behavioral therapy (CBT) has the most empirical support for treatment of BED.^8,9,10^ CBT focuses on disrupting the dietary restraint/binge eating cycle by encouraging regular eating and improving maladaptive thoughts and behaviors related to eating, shape, and weight. Although CBT reduces binge eating, it does not generally facilitate significant weight loss.^11,12,13^

The Behavioral Susceptibility Theory^14,15^ states that genetically determined appetitive traits influence how an individual interacts with the current obesogenic environment and highlights 2 mechanisms, eating onset and eating offset. Eating onset is driven by food responsiveness, defined as an individual’s sensitivity to environmental food cues, and eating offset is driven by satiety responsiveness, defined as an individual’s sensitivity to internal fullness sensations. We developed the regulation of cues (ROC) treatment to target both food responsiveness and satiety responsiveness. In a randomized clinical trial, we compared ROC, behavioral weight loss (BWL), ROC+BWL, and an active comparator (AC) among 271 adults with overweight or obesity. Participants randomized to ROC, ROC+BWL, and BWL had significantly lower body mass index (BMI; calculated as weight in kilograms divided by height in meters squared) after treatment and follow-up compared with those randomized to AC. Importantly, participants with higher food responsiveness lost more weight in ROC and ROC+BWL than in BWL or AC.^16^ All 4 groups showed significant reductions in binge eating severity, although the prevalence of binge eating was low.^17^ Data suggest that binge eating is associated with both food responsiveness and satiety responsiveness,^18,19,20,21,22,23^ leading to elevated cravings among individuals with binge eating.^21,24^ These mechanisms are targeted by ROC and could result in reduction of both binge eating and weight.

Rates of binge eating and overweight and obesity are high among veterans, with more than 65% of female and 45% of male veterans reporting symptoms of binge eating^25^ and 77% of veterans having overweight or obesity.^26,27^ Military experiences and norms (eg, eating quickly, periods of deprivation) put veterans at greater risk of binge eating.^28,29,30,31^ This study reports the main outcomes of a randomized clinical trial among veterans evaluating the impact of a 5-month ROC+BWL or CBT program on binge eating and overweight and obesity at the posttreatment assessment and the 6-month follow-up. We also evaluated the feasibility and acceptability of both treatments, the impact on energy intake, and whether binge eating moderated outcomes.

## Methods

The Controlling Hunger and Regulating Eating (CHARGE) randomized clinical trial was approved by the institutional review boards at University of California (UC) San Diego and the VA San Diego Healthcare System (VASDHS). All participants provided written informed consent. This study followed the Consolidated Standards of Reporting Trials (CONSORT) reporting guideline.

### Design

The CHARGE trial was a parallel randomized clinical trial that evaluated two 5-month treatments for veterans with binge eating and overweight or obesity: ROC+BWL and CBT. The study was conducted at UC San Diego between March 2019 and April 2023. Assessments were conducted at baseline, during treatment, posttreatment, and at 6-month follow-up assessments. Full trial design details have been published elsewhere,^32^ and the trial protocol and statistical analysis plan are provided in Supplement 1. Veterans were randomly assigned by the statistician using a permuted block randomization procedure^33^ to ROC+BWL or CBT stratified by sex and number of LOC episodes, with a block size ranging from 2 to 4.^34^ Outcome assessors were blinded to allocations until all assessments were completed. Data analysis was conducted with a limited dataset that only included participant identifiers and group allocations. The statistician had no contact with participants, interventionists, or assessors.

### Participants

Veterans were recruited from the VASDHS and the San Diego, California, metropolitan community. Recruitment targeted veterans interested in losing weight and managing their eating. Recruitment at VASDHS occurred through multiple clinics (eg, weight control, primary care, and mental health) using letters, flyers, tabling events, and physician referrals. The VASDHS team conducted a brief prescreen and reviewed VASDHS medical records, and veterans who appeared eligible provided written consent to share information with UC San Diego. Veterans from the community were recruited through flyers, email listservs, magazines for veterans, physician and study participant referrals, ClinicalTrials.gov, online recruitment advertisements, and tabling events.

Veterans were eligible if they (1) met *Diagnostic and Statistical Manual of Mental Disorders* (Fifth Edition) criteria for BED or subthreshold BED, (2) had BMI of 25 to 45, (3) were aged 18 to 65 years, and (4) did not meet any exclusion criteria (trial protocol in Supplement 1).^32^ Empirical power analysis supported the planned design of 60 participants per group,^26^ which would provide greater than 0.82 power to detect the expected treatment differences (approximately 5% decrease in BMI or reduction in binge eating episodes *d* = 0.50 based on meta-analyses)^11^ with allowance for up to 20% of participants lost to follow-up. Power analyses focused on individual outcome analyses and did not account for multiple outcomes.

### Intervention

Both treatments were provided in weekly 90-minute groups (18 sessions over 5 months) and provided the same goal of engaging in at least 250 minutes of moderate or vigorous intensity physical activity per week. Treatment for cohort 1 was provided in person and treatment for cohorts 2 through 5 was provided remotely via Health Insurance Portability and Accountability Act–adherent, password-protected video conference meetings (Zoom; Zoom Communications) due to COVID-19 restrictions.

### Treatment Groups

#### ROC+BWL

ROC+BWL included psychoeducation, self-monitoring, experiential learning, and coping skills.^16,35^ Psychoeducation focused on improving satiety responsiveness and decreasing food responsiveness and energy intake and included information about learning theory and the development of physiological responses to food cues; how appetitive traits can trigger overeating in today’s environment; how to use physiological, behavioral, and cognitive coping skills for mastery and tolerance of food responsiveness; and how to use energy calculations to make healthier food choices. Participants were taught to self-monitor hunger, cravings, food, calories, and physical activity on paper or using a smartphone application. Participants consumed meals during sessions and self-monitored their hunger on a 1 to 5 scale before, during, and after meals eaten and while simulating different contexts (eg, boredom, sadness). Participants also participated in exposures with highly craved foods during sessions and rated their cravings every 30 seconds on a 1 to 10 scale while holding, smelling, and tasting the food for 5 minutes. Participants were prescribed optimal calorie limits to target a 1- to 2-lb/week weight loss.

#### CBT

CBT followed an adapted manualized protocol.^36,37,38^ The first phase focused on reducing binge eating frequency by normalizing eating patterns to reduce dietary restraint, consuming regular meals and snacks, and incorporating a variety of foods into the diet. The second phase focused on addressing problematic cognitions related to eating behavior as well as shape and weight. The third phase focused on relevant topics, including self-esteem, body image, and problem-solving. Although information was provided about weight management in 1 session, weight loss was not emphasized, and caloric intake was not monitored. The final phase focused on reviewing progress and relapse prevention.

### Outcome Measures

Primary outcomes were feasibility, acceptability, changes in binge eating, BMI, and energy intake. Feasibility was measured with session attendance, and acceptability was measured with 15 questions that were rated on a 5-point Likert scale (eg, “I liked the CHARGE program overall,” and “The skills I learned in CHARGE were useful”). Full and subthreshold binge eating were measured by the Eating Disorder Examination (EDE) version 17.^34^ Full-syndrome BED included at least 12 objective binge eating episodes (OBEs; ie, a clinician-rated large amount of food with LOC) over the past 3 months. Subthreshold binge eating included: (1) at least 3 OBEs; (2) at least 6 subjective binge eating episodes (SBEs; ie, subjectively large amount of food accompanied by LOC) or LOC episodes; (3) at least 2 OBEs and 2 SBEs; or (4) at least 1 OBE and 4 SBEs over the past 3 months. The Binge Eating Scale (BES) was used to assess binge eating severity.^6,39^ Height was measured using a Seca 222 wall-mounted stadiometer, and weight was measured using a Tanita digital scale (model WB-380S). Height and weight were converted to BMI. After March of 2020, weight was measured using a Withings Bluetooth scale. Dietary intake was measured with three 24-hour dietary recalls^40,41,42^ on 3 nonconsecutive days. Physical activity was measured with the Global Physical Activity Questionnaire^43,44,45^ and was included as a covariate in all models. Demographics, including age, gender, and race and ethnicity, were self-reported via questionnaire. Participants were identified as Asian, Black or African American, Hispanic, White, multiple races, self-reported other race, or unreported. Race and ethnicity were assessed because there are expected differences among race and ethnicity groups in BMI, changes in BMI, and binge eating.

Participants received incentives for assessments at the following levels: $50 at baseline, $25 midtreatment, $100 posttreatment, and $150 at the 6-month follow-up. Participants in cohort 1 received $5 per group session attended to defray travel costs; however, when groups moved to remote delivery, this compensation was discontinued.

### Statistical Analysis

All analyses included an intention-to-treat approach, and missing data were evaluated prior to testing primary aims. All models included planned covariates (age, sex, race and ethnicity, LOC episodes, and physical activity) and corresponding baseline values. To assess feasibility, ROC+BWL was compared with CBT on treatment completion (defined as >70% of sessions) using logistic regression models. Between-group differences in acceptability were assessed with linear models. Models evaluating differences in binge eating episodes between ROC+BWL and CBT used mixed-effects ordinal logistic regression with a cumulative link. Models comparing effects of ROC+BWL and CBT on BMI and energy intake used linear mixed-effects models with bayesian joint imputation of missing data^46^ using R software version 4.4 (R Project for Statistical Computing). A dummy-coded index for each assessment was used given nonlinear patterns of change in outcomes, along with interaction terms evaluating between-group differences over assessments. Imputation models for normally distributed variables are specified as vague, inverse-γ distributions.^47^ Models estimated between-group point estimates for the adjusted odds ratio (AOR) of binge eating, tested whether the between-group estimates differed at assessments (eg, group by assessment), and then generated adjusted marginal estimates of differences in the probability of binge eating and mean differences in BMI reductions at planned midtreatment (2.5 months), posttreatment (5 months) and 6-month follow-up (11 months) assessments using planned covariates. In sensitivity analyses, divergence of estimates from imputed and complete-case models were evaluated and assumptions of missing at random for imputation models were evaluated by relating an index of missing assessments with demographic and available binge eating episodes outcomes. *P* values were 2-sided, and statistical significance was set at *P* ≤ .05. Data were analyzed from January 2024-June 2025.

## Results

### Participant Characteristics

We prescreened 1503 veterans from the community and 350 veterans from VASDHS and randomized 129 veterans (mean [SD] age, 47.1 [11.3] years; 76 [59%] male; mean [SD] BMI, 34.8 [4.7]), with 66 randomized to CBT and 63 to ROC+BWL (eFigure in Supplement 2). There were 6 Asian participants (5%), 7 Black or African American participants (5%), 42 Hispanic participants (33%), 63 White participants (49%), 7 participants (5%) who identified as multiple races, 2 participants (2%) who reported other race, and 2 participants (2%) with unknown race. A total of 39 participants (30%) had 12 or more OBEs in the last 3 months (Table 1). Randomization achieved group equivalence in these demographics. Five individuals started treatment but were excluded because of newly reported exclusionary preexisting medical conditions (eg, cancer). We used generalized estimating equations^48^ to evaluate the odds of missing data for assessments. At the midtreatment assessment, 6 participants (5%) had missing data; at the posttreatment assessment, 7 participants (5%) had missing data; and at the 6-month follow-up, 14 participants (11%) had missing data. Odds of completed postrandomization binge eating and BMI assessments did not differ across treatments (OR, 0.73; 95% CI, 0.45-1.18; *P* = .19).

**Table 1.** Demographics of the Sample

Characteristic	Participants, No. (%)		
	CBT (n = 66)	ROC+BWL (n = 63)	Total (n = 129)
Age, mean (SD)	46.3 (11.2)	47.9 (11.4)	47.1 (11.3)
Sex			
Male	39 (59)	37 (59)	76 (59)
Female	27 (41)	26 (41)	53 (41)
Race and ethnicity			
Asian	4 (6)	2 (3)	6 (5)
Black or African American	3 (5)	4 (6)	7 (5)
Hispanic	23 (35)	19 (30)	42 (33)
Multiple races	5 (8)	2 (3)	7 (5)
Reported other	1 (2)	1 (2)	2 (2)
Unreported	0	2 (3)	2 (2)
White	30 (45)	33 (52)	63 (49)
Branch of military			
Air Force	7 (11)	3 (5)	10 (8)
Army	16 (24)	11 (17)	27 (21)
Coast Guard	0	1 (2)	1 (1)
Marine Corps	6 (9)	8 (13)	14 (11)
Navy	36 (54)	34 (54)	70 (55)
Multiple branches	1 (2)	5 (8)	6 (5)
Not reported	0	1 (2)	1 (1)
Combat exposure			
No	43 (65)	37 (59)	80 (62)
Yes	23 (35)	25 (40)	48 (38)
Missing	1 (1)	0	1 (1)
BMI, mean (SD)	34.7 (5.0)	34.9 (4.4)	34.8 (4.7)
Loss of control episodes, median (IQR), No.^a^^,^^b^	33 (16-56)	32 (15-59)	32 (16-58)
Objective binge episodes, No.^b^			
≥12	22 (33)	17 (27)	39 (30)
<12	44 (67)	46 (73)	90 (70)
BES, mean (SD) score	21.3 (7.1)	19.7 (7.8)	20.0 (5.9)
MVPA per wk, median (IQR), min	285 (112-848)	360 (94-825)	312 (105-832)

Abbreviations: BES, Binge Eating Scale; BMI, body mass index (calculated as weight in kilograms divided by height in meters squared); BWL, behavioral weight loss; CBT, cognitive behavioral therapy; MVPA, moderate to vigorous physical activity; ROC, regulation of cues.

^a^ Used for randomization.

^b^ Past 3 months.

### Feasibility and Acceptability

Both treatments were well attended, with a median of 15 sessions attended in both ROC+BWL (IQR, 13-17 sessions) and CBT (IQR, 11-17 sessions). Logistic regression with adjustment for planned covariates did not support a difference in the percentage of participants who attended more than 70% of sessions for ROC+BWL or CBT (AOR, 1.52; 95% CI, 0.66-3.57; *P* = .40). Rates of attendance were not significantly associated with any covariates. Participants generally liked both treatments similarly, and rated agree (median [IQR] score, 3.3 [2.9-3.6]) on the acceptability and satisfaction posttreatment questions (coefficient α = 0.93). There were no differences in mean ratings between ROC+BWL and CBT (adjusted mean difference, 0.14; 95% CI, −0.09 to 0.37; *P* = .23).

### Binge Eating

**Figure 1.**
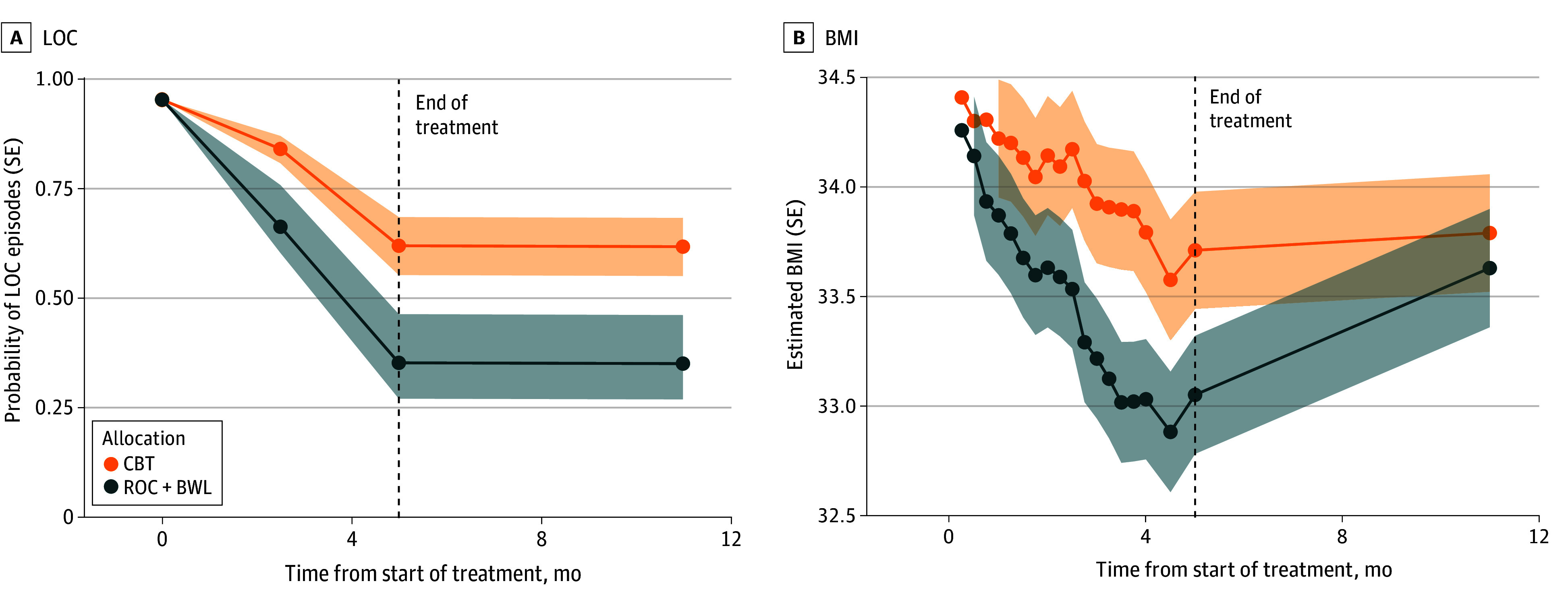
Changes in the Probability of Loss of Control (LOC) Eating Episodes and Body Mass Index (BMI) in Regulation of Cues (ROC) With Behavioral Weight Loss (BWL) and Cognitive Behavioral Therapy (CBT) Groups Over the Trial

ROC+BWL resulted in a greater reduction in risk of binge eating than CBT at midtreatment (difference in probability, −0.20; 95% credible interval [CrI], −0.30 to −0.11), posttreatment (difference in probability, −0.23; 95% CrI, −0.22, −0.19) and at the 6-month follow-up (difference in probability, −0.21; 95% CrI, −0.21 to −0.18) (Figure 1 and Table 2). Participants in both groups decreased their LOC episodes, from 58 participants in each group at baseline to 26 participants (45%) for ROC+BWL and 37 participants (64%) for CBT at the posttreatment time point (Table 2). The covariate-adjusted model supported a difference in LOC for ROC+BWL compared with CBT and the size of the difference between groups did not differ across assessments (eTable in Supplement 2).

**Table 2.** Changes in the Probability of LOC Episodes for ROC+BWL vs CBT at Each Assessment^a^

LOC episodes, No.^b^	CBT (n = 66)		ROC+BWL (n = 63)		Difference in probability (95% CrI)	*P* value
	No. (%)	Probability (95% CrI)	No. (%)	Probability (95% CrI)		
Midtreatment						
0	9 (15)	0.11 (0.04 to 0.20)	18 (30)	0.30 (0.15 to 0.50)	−0.20 (−0.30 to −0.11)	<.001
1-2	9 (15)	0.11 (0.05 to 0.19)	8 (13)	0.21 (0.14 to 0.28)		
3-9	13 (21)	0.32 (0.22 to 0.41)	20 (33)	0.31 (0.20 to 0.40)		
≥10	30 (49)	0.47 (0.27 to 0.68)	14 (23)	0.19 (0.08 to 0.34)		
Missing, No.	5	NA	3	NA	NA	NA
Posttreatment						
0	21 (36)	0.36 (0.19 to 0.56)	32 (55)	0.59 (0.38 to 0.79)	−0.23 (−0.22 to −0.19)	.005
1-2	10 (17)	0.21 (0.15 to 0.28)	7 (12)	0.18 (0.11 to 0.26)		
3-9	14 (24)	0.28 (0.17 to 0.38)	12 (21)	0.16 (0.07 to 0.28)		
≥10	13 (22)	0.15 (0.06 to 0.28)	7 (12)	0.06 (0.02 to 0.14)		
Missing, No.	8	NA	5	NA	NA	NA
6-mo follow-up						
0	20 (39)	0.37 (0.19 to 0.58)	31 (56)	0.59 (0.37 to 0.79)	−0.21 (−0.21 to −0.18)	.01
1-2	9 (18)	0.21 (0.15 to 0.28)	7 (13)	0.18 (0.11 to 0.26)		
3-9	10 (20)	0.28 (0.16 to 0.38)	7 (13)	0.16 (0.07 to 0.28)		
≥10	12 (24)	0.14 (0.05 to 0.27)	10 (18)	0.06 (0.02 to 0.13)		
Missing, No.	15	NA	8	NA	NA	NA

Abbreviations: BWL, behavioral weight loss; CBT, cognitive behavioral therapy; CrI, credible interval; LOC, loss of control episode; NA, not applicable; ROC, regulation of cues.

^a^ Model estimates adjusted age, sex, race and ethnicity, body mass index at baseline, LOC episodes at baseline, and physical activity.

^b^ Difference in probability of LOC more than 0; covariate adjusted probability of 1 or more binge episodes for ROC+BWL compared with CBT at each assessment.

Mean BES scores did not significantly change in ROC+BWL compared with CBT at the posttreatment assessment (between-group difference, −2.66; 95% CI, −0.97 to 6.28) and at the 6-month follow-up assessment (between-group difference, −1.97; 95% CI, −2.01 to 6.01). Both groups had large decreases in BES scores from a moderate severity range at baseline to a range considered low severity at posttreatment, from a mean (SD) score of 19.66 (7.79) at baseline to 8.22 (7.46) at posttreatment in the in the ROC+BWL group and 21.27 (7.05) at baseline to 11.91 (7.83) at posttreatment in the CBT group. The covariate-adjusted model supported the presence of differences in BES for ROC+BWL compared with CBT; however, the differences were not statistically significant, so we could not reject the hypothesis of no differences (eTable in Supplement 2).

### BMI

Veterans in ROC+BWL had greater weight loss than the CBT group at the midtreatment (difference in BMI change, −0.68; 95% CrI, −1.23 to −0.12) and posttreatment (difference in BMI change, −0.71; 95% CrI, −1.40 to −0.01) assessments; however, significant differences were no longer observed at the 6-month follow-up (difference in BMI change, −0.22; 95% CrI, −0.98 to 0.54) (Table 3). Both ROC+BWL and CBT treatments resulted in decreased BMI across the study (Figure 1). The covariate-adjusted model supported greater reductions in BMI for ROC+BWL compared with CBT, and the size of the between-group effects were different across assessments (eTable in Supplement 2).

**Table 3.** Changes in Mean BMI for ROC+BWL vs CBT at Each Assessment

Assessment	ROC+BWL			CBT			Difference in change^a^	
	No.	BMI		No.	BMI		Estimate (95% CrI)	*P* value
		Mean (SD)	Estimate (95% CrI)^b^		Mean (SD)	Estimate (95% CrI)^b^		
Session 1	63	34.25 (4.15)	34.30 (33.70 to 34.80)	66	34.51 (4.83)	34.40 (33.90 to 34.90)	NA^b^	NA^b^
Midtreatment	60	33.81 (4.22)	33.50 (33.00 to 34.10)	63	34.41 (5.21)	34.20 (33.60 to 34.70)	−0.68 (−1.23 to −0.12)	.02
Posttreatment	61	33.41 (4.53)	33.10 (32.50 to 33.60)	61	33.73 (5.24)	33.70 (33.20 to 34.20)	−0.71 (−1.40 to −0.01)	.048
6-mo follow-up	58	33.89 (4.64)	33.60 (33.10 to 34.20)	57	33.96 (5.49)	33.80 (33.30 to 34.30)	−0.22 (−0.98 to 0.54)	.58

Abbreviations: BMI, body mass index (calculated as weight in kilograms divided by height in meters squared); BWL, behavioral weight loss; CBT, cognitive behavioral therapy; CrI, credible interval; NA, not applicable; ROC, regulation of cues.

^a^ Model estimates adjusted age, sex, race and ethnicity, BMI at baseline, loss of control episodes at baseline, and physical activity.

^b^ Adjusted BMI estimate from joint imputation model.

^c^ Session 1 was the reference assessment in models, so there is no between-group evaluation and no-tail *P* value.

### Energy Intake

Participants in ROC+BWL reported greater reduction in caloric intake than those in CBT at posttreatment (difference, −280.16 [95% CrI, −446 to −113] kcal) and 6-month follow-up (difference, −300.00 [95% CrI, −465 to −133] kcal) assessments. The median (IQR) change in caloric intake for ROC+BWL and CBT treatments was −392.70 (−792.60 to 19.90). The covariate-adjusted model supported a greater reduction in caloric intake for ROC+BWL compared with CBT, and the size of the between-group effects were not different across assessments (eTable in Supplement 2).

### BED as a Moderator of Treatment Outcomes on Binge Eating and BMI

**Figure 2.**
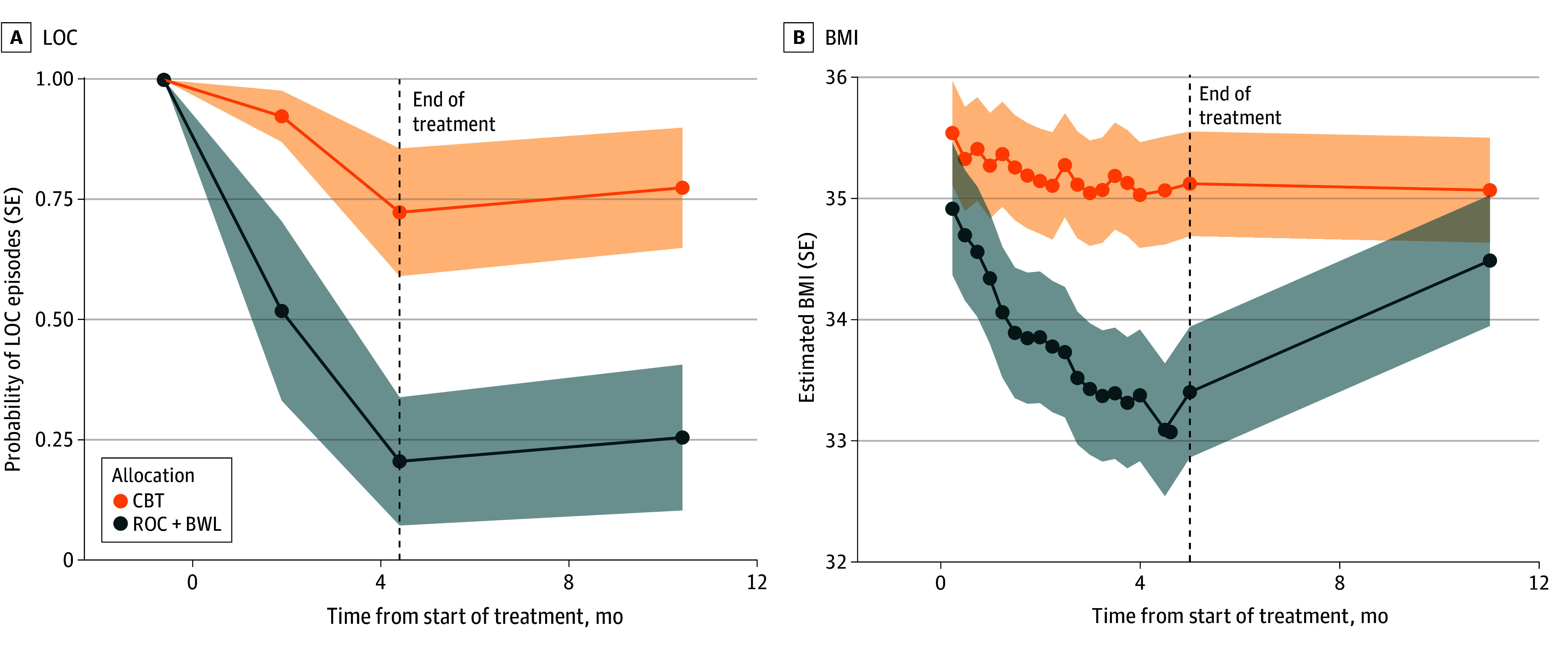
Moderating Effect of Full Syndrome Binge Eating Disorder on Loss of Control (LOC) Eating Episodes (n = 37) and Body Mass Index (BMI) (n = 39) Over the Trial

The beneficial effects of ROC+BWL vs CBT on LOC at midtreatment, posttreatment and 6-month follow-up assessments were stronger for participants entering treatment with BED compared with the subthreshold binge eating group (Figure 2). Follow-up stratified models examining the BED group supported a larger difference when comparing ROC+BWL vs CBT (AOR, 0.07; 95% CI, 0.01 to 0.36; *P* = .002) than for the subthreshold binge eating group (AOR, 0.50; 95% CI, 0.20 to 1.18; *P* = .04). The beneficial effects of ROC+BWL vs CBT on BMI at the midtreatment and posttreatment assessments were also stronger in participants with BED compared with the subthreshold binge eating group (Figure 2). In stratified models, greater BMI decreases for ROC+BWL vs CBT from the midtreatment through posttreatment assessments ranged from −0.92 to −1.35 for those with BED. However, BMI for ROC+BWL was no longer significantly different from CBT at the 6-month follow-up assessment in the BED sample or for those with subthreshold binge eating in covariate adjusted models (eTable in Supplement 2).

## Discussion

In this randomized clinical trial among veterans with binge eating and obesity, we found that both ROC+BWL and CBT were similarly feasible and acceptable and that participants in ROC+BWL had greater decreases in binge eating episodes, weight, and caloric intake during treatment compared with those in CBT. The reduction of binge eating episodes and caloric intake for ROC+BWL participants were maintained at the 6-month follow-up, but differences in weight were not maintained.

ROC+BWL was more effective at reducing binge eating frequency (as measured by the EDE)^34^ throughout the study, which is remarkable because meta-analyses show that CBT is effective at reducing binge eating in the short term but not the long term.^49^ The differences in binge eating frequency was more robust when evaluating individuals with BED but did not reach statistical significance in individuals with subthreshold BED and needs to be confirmed in larger samples. These differences could be attributed to the different mechanisms targeted in ROC+BWL and CBT. CBT targets top-down changes in behaviors and cognition and focuses on reducing dietary restraint and decreasing overevaluation of weight and shape. ROC+BWL targets both top-down and bottom-up mechanisms by training the use of appetitive cues, inhibition of urges to eat when not physically hungry, and toleration of cravings and food restriction. It is possible that targeting both top-down and bottom-up mechanisms could provide a more effective and durable treatment for binge eating. Notably, binge eating severity was not different between ROC+BWL and CBT, although there were differences in LOC using the EDE interview. This discrepancy could suggest that ROC+BWL was more effective at reducing binge eating frequency but not severity. This discrepancy could also be due to the measurement of LOC using the EDE interview,^34^ compared with the measurement of binge eating severity using a self-report questionnaire (BES).^6,39^

ROC+BWL also outperformed CBT in terms of weight loss during treatment. Similar to previous interventions that include caloric restriction, we saw weight rebound in ROC+BWL during the 6-month follow-up, with no significant differences in weight loss between ROC+BWL and CBT in either the entire sample or those with full-syndrome BED. However, the dietary intake data suggested that participants in ROC+BWL decreased their daily caloric intake more than participants in CBT at posttreatment and 6-month follow-up assessments. Weight loss and maintenance in CBT was unexpected, as most data suggest that participants in CBT do not lose significant amounts of weight, but there is wide variability.^11,12,13^ For example, in a study among 189 adults treated with CBT, mean weight change was +1.3 lbs, and 22% of the sample lost more than 5 lbs and 25% of the sample gained more than 8 lbs.^13^ The weight loss in CBT in this trial could be due to increased adherence to treatment recommendations among veterans due to their history of military training. We also included a session on weight loss in CBT, as was included in the manual.^37^ There also could have been a bias to change eating behavior and weight, since the advertisements targeted both binge eating and weight loss. Finally, it is also possible that participants in either group engaged in other weight management behaviors that were not reported.

To our knowledge, this is the first study to show that another model, ROC+BWL, was more effective at treating binge and LOC eating than CBT, and these effects were more pronounced among individuals with full-syndrome BED. To our knowledge, the only other model that has been directly compared with CBT is interpersonal psychotherapy, with studies showing equivocal results.^32,43,44^ In this study, we chose to use ROC+BWL, which includes energy restriction, instead of the ROC model that only focuses on targeting food responsiveness and satiety responsiveness.^16,50^ This decision was made before the results of our previous clinical trial were known.^16^ In that trial, participants in the ROC+BWL group had greater weight loss but similarly had a rebound in weight, whereas participants receiving ROC alone had a slower rate of weight loss but did not see a rebound. We were unable to evaluate binge and LOC eating rates in that study, as the rates were too low in that sample. It is possible that the ROC model alone without dietary restriction may have been as effective or more effective for veterans, a question that needs to be explored in future clinical trials. It is also possible that participants overly focused on calorie counting as opposed to monitoring their hunger and responsiveness to food cues and ultimately did not learn the unique ROC skills. Importantly, given the concerns about weight cycling and mental health^51,52^ and to provide a precision medicine approach, it is important to find a treatment that both can reduce binge eating and maintain weight loss that is specifically tailored to the individual.

Strengths of the study include the evaluation of a novel intervention, ROC+BWL, compared with an empirically supported treatment for binge eating, CBT, among a sample of veterans. This was a diverse sample of treatment-seeking veterans that was balanced on sex, which is unusual for studies of binge eating and weight.

### Limitations

This study has some limitations. Limitations include the self-reported nature of assessments for binge eating, dietary intake, and physical activity, meaning they are subject to bias. Additionally, we only assessed weight using BMI, and it is possible that the treatments differentially affected other metabolic indicators. Because it was a treatment-seeking sample, these results cannot be generalized to the general population of veterans or individuals in the community. Power analyses that focused on binge eating and BMI outcomes did not account for multiple testing and may have resulted in underpowered evaluations. Decisions to limit corrections for multiple testing may increase risk of incorrectly identifying between-group differences, and replication is needed to ensure the robustness of our conclusions. Furthermore, as there were only 6 months of follow-up, we cannot determine the durability of these treatment effects over time. Future studies with longer follow-up in veteran and community samples are needed.

## Conclusions

The findings of this randomized clinical trial have clinical implications for the treatment of veterans who have binge eating and obesity. Based on these results, ROC+BWL could provide an alternate model for the treatment of both binge eating and obesity in veterans, in particular for those with full-syndrome BED. ROC+BWL targets both appetitive traits and reduction in energy intake, which could provide multiple distinct skills to manage urges to binge eat and overeat and provide a more durable treatment. This study also highlights the potential for targeting appetitive traits to change eating behavior. Although this investigation provides initial support for the use of ROC+BWL as an alternative model to treat binge eating among veterans, more research is needed on the effects on weight and on these treatments among community samples, and longer-term studies are needed, as well as studies that include ROC alone.

## Notes

Supplement 1.
Trial Protocol and Statistical Analysis Plan

Supplement 2. **eTable 1.** Differences Between ROC+BWL and CBT on Loss of Control, Binge Eating Severity, Body Mass Index, and Energy Intake **eFigure.** Trial Recruitment Flowchart

Supplement 3.
Data Sharing Statement
