# Supplementary material for: Regulation of Cues vs Cognitive Behavioral Therapy for Binge Eating and Weight Loss Among Veterans: A Feasibility and Randomized Clinical Trial
Source: JAMA Netw Open. 2025 Aug 4;8(8):e2525064. doi: 10.1001/jamanetworkopen.2025.25064 (PMC12322792; doi:10.1001/jamanetworkopen.2025.25064)
Supplement: Supplement 1. — Trial Protocol and Statistical Analysis Plan [file jamanetwopen-e2525064-s001.pdf]

UCSD Human Research Protections Program
New Biomedical Application
RESEARCH PLAN

Instructions for completing the Research Plan are available on the HRPP website.
The headings on this set of instructions correspond to the headings of the Research Plan.
General Instructions: Enter a response for all topic headings.
Enter "Not Applicable" rather than leaving an item blank if the item does not apply to this project.

Version date: 9/30/2013

1. PROJECT TITLE

Targeting Food Cue Reactivity and Satiety Sensitivity to Decrease Binge Eating and Weight

For participants enrolled in the study we will call the study CHARGE – Controlling Hunger and ReGulating Eating
The official title will be used in correspondence with the IRB

2. PRINCIPAL INVESTIGATOR

Kerri Boutelle, Ph.D,

3. FACILITIES

UC San Diego Center for Healthy Eating and Activity Research (CHEAR)
La Jolla Village Professional Center
8950 Villa La Jolla Drive, Suite C-203
La Jolla, CA 92037

VA San Diego Healthcare System (reference IRB approval will be obtained at this site.) (VASDHS)
3350 La Jolla Village Dr
San Digo, CA 92161

4. ESTIMATED DURATION OF THE STUDY

4 years

5. LAY LANGUAGE SUMMARY OR SYNOPSIS (no more than one paragraph)

This application will test a novel treatment (Regulation of Cues; ROC) for Veterans with both binge eating disorder (BED) and overweight/obesity (OW/OB), which unlike current gold-standard interventions for BED, could have a significant clinical impact on both binge eating and weight. This proposal is a randomized controlled trial evaluating ROC and cognitive behavioral therapy (CBT) on reduction of binge eating and weight loss among Veterans. 120 male and female Veterans with BED or subclinical BED with comorbid OW/OB will be randomized to 5-month ROC or CBT treatment and will be followed for 6-months post-treatment. Participants will complete assessments at baseline, mid-treatment (~week 10), post-treatment (week 20), and 6-month follow-up (week 44). Participants will complete brief survey assessments monthly during treatment to assess mediators. We will also collect data on feasibility and acceptability of the two treatments.

6. SPECIFIC AIMS

Primary Aim 1: Evaluate the feasibility and acceptability of ROC and CBT.

Primary Aim 2: Compare the efficacy of ROC and CBT on binge eating over the duration of the study.

Primary Aim 3: Compare the efficacy of ROC and CBT on weight loss (BMI) and energy intake (as measured by 24-hour dietary recalls) over the duration of the study.

Secondary Aim 1: Determine the extent to which ROC and CBT influence underlying mechanisms of action, including responsivity to food cues, satiety responsiveness, and reward-based eating.

Exploratory Aim 1: To evaluate moderators of treatment (age, gender, binge eating status, satiety responsiveness, responsivity to food cues, reward-based eating) and mediators of treatment outcomes (responsivity to food cues, satiety responsiveness, reward-based eating, physical activity).

7. BACKGROUND AND SIGNIFICANCE

Cognitive behavior therapy (CBT) is considered the gold standard for the treatment of Binge eating disorder (BED).[1] CBT focuses on disrupting the restraint/binge cycle by improving maladaptive thoughts surrounding eating,

shape, and weight, and encouraging healthy weight control behaviors. Participants in CBT, compared to waitlist controls, achieved abstinence from binge eating and reported improved eatin g-related psychopathology.[2] However, CBT fails to produce significant weight loss.[2,3] Theoretically, if binges are decreased, individuals should lose weight if they decrease binge eating. CBT assumes that individuals can access and consciously control cognitions that maintain BED. However, dual process theories suggest that implicit processes (i.e. food cue responsivity) may also exert control over behavior.[4] Addressing implicit processes could enhance treatments for BED and result in reductions in binge eating and enhanced weight loss.

Dr. Boutelle developed a treatment program that targets improved appetite sensitivity and decreased food cue responsivity, called Regulation of Cues (ROC). She implemented ROC among adults who binge eat with OW/OB. Data showed that there was a significant weight loss and reduction of bing e eating post-treatment and three months post-treatment.[5] Dr. Boutelle is also utilizing the ROC treatment in another study entitled "Treatment of obesity targeting appetite and cue reactivity" #151110 for adults with OW/OB.

A logical next step in the development of the ROC program is to validate the protocol with adults with BED. The premise of this proposed project is to comparing the ROC intervention to CBT for Veterans with BED or subclinical BED.

8. PROGRESS REPORT

This is a new application

9. RESEARCH DESIGN AND METHODS

DELEGATION OF RESPONSIBILITIES

University of California, San Diego (UCSD) and the Veteran Affairs San Diego Healthcare System (VASDHS) will be collaborating on this study. This study will include two consent forms; One for UCSD, and one for VASDHS. All participants will complete the UCSD consent form (consented by UCSD-approved UCSD study staff). However, only the subset of participants who are recruited directly from the VA with VA study staff will complete the VASDHS consent form (consented by VASDHS study staff). See additional details under "Section 11.RECRUITMENT AND PROCEDURES PREPARATORY TO RESEARCH" and "Section 12 INFORMED CONSENT"

The Collaborators agree to perform the following tasks:

VASDHS Staff will be responsible for the following tasks under VASDHS IRB approval:

- Obtaining VASDHS Institutional Review Board (IRB) approval of all study related documents and subsequent continuing reviews.
- Study recruitment and determining preliminary eligibility for the participants recruited directly from VASDHS
- Obtaining informed consent and HIPAA authorization for screening from all VASDHS-recruited study participants using VASDHS IRB approved forms.
- Collaboratively generating manuscripts based on study findings.

UCSD staff will be responsible for the following tasks under UCSD IRB approval (Kerri Boutelle, PhD, Principle Investigator):

- Hiring and training UCSD study staff for the conduct of the study.
- Obtaining UCSD IRB approval of all study related documents and subsequent continuing reviews.
- Study recruitment and determining preliminary eligibility for the participants recruited in the community.
- Obtaining informed consent from all study participants using UCSD IRB approved forms.
- Delivering the intervention.
- Conducting data collection, including administration of self-reported assessments and assessor Administered assessments at all assessment time points.
- Serving as the coordinating center and overall study monitor, to include collection of all study data for analysis and storage at UCSD.

Research Design and Methods

Study Overview. This proposal is a randomized controlled study with two arms: ROC and CBT. The ROC program targets improved satiety responsiveness and decreased food responsiveness and provides psychoeducation (ways the environment "tricks" the body into overeating past nutritional needs, coping skills designed to assist in mastery and toleration of food cue sensitivity, daily self-monitoring (hunger, satiety, and cravings)) and experiential learning (exposure sessions while self-monitoring hunger, satiety, and cravings). Additionally, the ROC participants will be taught to decrease caloric intake and increase physical activity, to assist with satiety responsiveness and to decrease food cue responsiveness. The CBT treatment program focuses on disrupting the restraint/binge cycle by improving maladaptive thoughts surrounding eating, shape, and weight. As part of CBT, participants self-monitor food intake, to understand the thoughts that trigger binge eating and as a way to identify times of restriction that can trigger binge eating. CBT targets explicit urges to binge eat but does not include experiential exercises. (See Table 1)

We will recruit 300 overweight and obese male and female Veterans with BED or subclinical BED to randomize at least 120 meeting all inclusion criteria, will provide 5 months of treatment and will follow participants for 6-months post-treatment. Total length of participation will be approximately 11 months. Participants will complete assessments at baseline, mid-treatment (~week 10), post-treatment (week 20), and 6-month follow-up (week 44). Participants will complete brief survey assessments monthly during treatment to assess mediators. We will also collect data on feasibility and acceptability of the two treatments. In person assessments will take 3-4 hours except for the mid treatment which will be about 1 hour. This study is being conducted in collaboration with investigators from the VA San Diego Healthcare System (VASDHS). The collaborators will file a separate IRB proposal through the VA to cover their research activities which will include directly recruiting Veterans from health clinics at the VA and conducting the initial screen. Information collected in their screen will securely be shared with our research team. UCSD team will complete the remainder of screening, assessments and treatment of the participants.

Research Design. This study will randomize at least 120 overweight and obese male and female Veterans with BED or subclinical BED to one of two arms (ROC or CBT). The study data manager, who will have no contact with research participants, will conduct randomizations and monitor allocations throughout the study to alert the PI of any systematic differences in additional demographics (age, race/ethnicity) that may arise.

Treatment Arms

Both interventions arms will be matched on contact frequency and time. All treatment groups will include up to 20 participants, will be 60 minutes in length and will meet weekly for 4 months, and twice monthly for the 5th month (total treatment duration = 5 months, 18 meetings). Descriptions of the treatment arms follow and key differences between the treatment arms are outlined in Table 1. All treatment sessions will be audiorecorded for supervision and fidelity purposes.

Table 1. Differences between ROC and CBT

	ROC	CBT
**Dietary prescription**	Sessions focus on learning to control physiological and psychological responding to food, and to eat less of foods that are palatable when physically sated. Participants will learn general food intake literacy, to pick nutritious foods, and physiological energy needs.	None Participants are encouraged to incorporate a variety of foods into their diet
**Self-monitoring**	Hunger, craving and food intake	Antecedents, behaviors, consequences and thoughts surrounding binge episodes
**Experiential learning**	Participants bring meals and/or palatable foods to each session. Hunger and satiety is monitored in session during meals. Exposures to highly craved foods are conducted in session.	None
**Physical activity prescription**	Physical activity is used to help regulate physiological and psychological responding to food cues.	None
**Goal setting**	Goal setting focuses on self-monitoring and practicing mastery and toleration of physiological /psychological arousal, as well as food intake and physical activity	Goal setting focuses on self-monitoring of food intake, thoughts and behaviors.
**Coping skills**	Methods for managing psychological and physiological arousal. Discussed each week in regards to mastery and toleration of physiological /psychological arousal.	Cognitive restructuring, stress management, behavior chains to reduce binge eating

Regulation of Cues (ROC). The ROC program provides psychoeducation, coping skills, self-monitoring and experiential learning. Physical activity will be promoted to improve self-regulatory strength and to help participants master and tolerate physiological and psychological arousal, resist cravings and overeating.

Psychoeducation. The ROC program provides psychoeducation at each group visit by describing a "Deceptive Hunger", which is a way that the environment "tricks" the body into overeating past nutritional needs. The overall goal of psychoeducation is to increase participant's awareness of the reasons why they may overeat, and to relieve participants from guilt regarding overeating by helping them understanding the processes by which these phenomena occur. Both lack of sensitivity to appetite and satiety cues and increased sensitivity to food cues will be discussed. Physiological, neurobiological and environmental models of overeating past nutritional needs are presented in lay language so that participants can understand their vulnerabilities to overeating. The concepts are taught using a chronic disease model in which the individual is considered to have the biological vulnerability to overeat that is amplified by the current obesogenic food environment. Participants are provided information about basic learning theory and how physiological responses to food cues develop and can be managed.

Coping skills. Coping skills will be taught to accompany each Deceptive Hunger. Coping skills are presented to assist in mastery and toleration of food cue sensitivity. Coping skills include physiological skills (deep breathing, relaxation, and mindfulness), behavioral skills (delay, activity substitution) and cognitive skills (cognitive restructuring, distraction).

Experiential learning and self-monitoring. In each session, participants will complete an experiential learning exercise. During visits 1-8, participants will be taught about hunger and satiety dysregulation. Participants will be taught to monitor their hunger in a self-monitoring booklet on a 1-5 scale, with 1 “starving” and 5= “stuffed”. Participants will be instructed to self-monitor hunger and satiety before, during and after each meal, as well as 10 and 20 minutes after eating for a minimum of two meals/snacks per day. Participants will bring dinn er and all groups will start by eating dinner and monitoring their hunger with prompting from the group leader.

During visits 9-16, participants will learn to assess and rate their cravings (defined as urges to eat when not physically hungry). Craving is monitored with a 5-point scale, 1= “not craving it at all” and 5=”craving is overwhelming” and participants will rate cravings during the day (ideally one craving a day at minimum). Participants will create a craving hierarchy and will bring their highly craved foods to group and will complete an exposure at each session (CET-Food). Exposures are only conducted when participants are not physically hungry. If a participant is physically hungry, they will have a snack before participating in an expos ure. During the exposure, participants will rate their cravings while looking at the food, holding the food, smelling the food, after taking two small bites of the food, and then will rate their cravings at 30-second intervals for the duration of the exposure. After 10 minutes, the participants dispose of the food without eating it and the exposure ends.
In all the following weeks participants will monitor both their hunger and cravings.

Cognitive Behavior Therapy (CBT). CBT is the current standard treatment for BED. CBT includes three phases and is founded on the concept that binge eating patterns develop as a response to repeated restrictive dieting. This is described as a binge cycle, which begins with distorted attitudes a bout shape and eating and pressure to be thin, which leads to excessive restrictive dieting, which leads to feeling hungry and deprived, inevitably leading to binge eating episodes, triggering negative thoughts about oneself which starts more restriction a nd the cycle is repeated.

The initial focus in CBT is on eliminating binge eating patterns by establishing regular, healthy eating patterns. Participants are taught to self-monitor their food intake and eating patterns, binge episodes, thoughts, and mood pre- and post- binge, as well as the circumstances and environment surrounding eating. Examination of the monitoring records are the primary focus of the group treatment. Participants are taught to identify problems in eating, thinking, and mood patterns that served to trigger binge episodes through self-monitoring and to gradually develop alternative patterns aimed at facilitating healthy, binge-free eating patterns. Participants are taught to eat a healthy variety of foods, and later on in treatment, they are taught to integrate the binge foods into their usual meals. CBT treatment consists of three phases.

In the first phase, focus is on reducing binge eating by normalizing eating patte rns to reduce dietary restraint and incorporating a variety of foods into the diet. In the second phase, focus is on addressing problematic cognitions related to eating shape and weight. Further, emphasis is placed on identifying alternative responses to stress than binge eating. Finally, in the third phase, participants are instructed to anticipate high-risk situations in which a lapse might occur and to outline a plan to prevent such a lapse as well as develop long-term realistic expectations for their eating habits.

Quality control, fidelity and supervision of intervention. The intervention for this study will be provided by licensed clinical psychologists and postdoctoral fellows. All interventionists in ROC will attend a 2-day training with Dr. Boutelle and Dr. Bernard and all interventionists in CBT will attend a 2-day training with Dr. Peterson and Dr. Bernard. Intervention sessions will be audiotaped for ongoing performance monitoring. Random samples (e.g. 30% or more) of all intervention sessions will be rated for fidelity by an independent rater using a measure created for this study.

Study Assessment Procedures:
As detailed in section 12, prescreening for participants from the community will be obtained online and via the phone by UCSD staff. Prescreening for participants coming through the VA will be conducted by VA personnel and covered under the VASDHS IRB.

Participants deemed eligible by the prescreening from either UCSD or VA staff will be invited to an orientation visit at CHEAR. A summary of the in-person visits at UCSD are provided below and descprition of the measures administred follow.

Orientation Visit:
At this visit, UCSD study staff will again describe the study, the two treatments arms, randomization methods, expectations for participation, and incentives for time and effort to ensure participants completely understand what they are signing up for and be presented with the informed consent. Participants who agree to participate and complete the consent form will have their height and weight measured and schedule their baseline assessment visit.

Baseline Visit
This visit will take 3-4 hours and consist of completing some interviews and quesitonnaires for eligibility and other anthropometric measures, tasks, and questionnaires to collect baseline data. After the visit and prior to starting treatment, participants will complete the dietary recalls over three differnet phone calls on 3 different days.

Participants who complete the Baseline assessment procedures and remain eligible will be randomized about a week before treatment is scheduled to start and then will start treatment

Mid-Treatment Visit
Around week 10 of treatment, participants will attend another in-person assessment (approximately 1 hour) to complete some interviews and questionnaires completed during the baseline visit.

Post Treatment and 6-month Follow-up Visit
Participants will complete two more visits (~3-4 hours), one immediately after treatment and one approximately 6 months after treatment ends to complete interviews, surveys, tasks. Participants will again be asked to complete dietary recalls on the phone outside of the visit.

Measures
A complete summary of the list of assessment measures and time points administered are provided in Table 2. The primary outcome measures are provided in more detailed below.

Psychiatric symptoms (baseline only). Participants will complete the Mini-International Neuropsychiatric Interview (MINI[6] to confirm eligibility at baseline (i.e., absence of exclusionary psychiatric condition). The MINI 7.0 is a short, structured diagnostic interview developed to assess psychiatric disorders according to DSM-5 criteria, focusing on current symptomatology.

Medical diagnoses and medications. Participants will report any known medical diagnoses and current and past medications to confirm eligibility at baseline. We will follow-up monthly during treatment to see if any changes have occurred and again at follow-up.

Demographics and military characteristics (baseline only). Participants will complete a demographic questionnaire to record their age, gender, years of education, socioeconomic status, branch of service, years and position in military, highest rank at discharge, and years since actively serving in the military.

The Eating Disorder Examination (EDE) interview [7](baseline, mid-treatment, post-treatment, 6-month follow-up) is considered the gold-standard assessment for BED and has strong psychometric properties. The EDE interview uses diagnostic algorithms to diagnose BED, as well as subclinical BED, frequency of binge eating, compensatory behaviors (purging for exclusion), as well as restraint and shape and weight concerns.

The Binge Eating Scale[8] (BES) (*baseline, mid-treatment, post-treatment, 6-month follow-up)* provides a dimensional score of binge eating. The BES consists of 16 items; 8 items describe binge eating behaviors and 8

describe feelings and cognitions associated with binge eating. The BES yields a continuous measure of binge eating pathology (range=0–46).

The Eating Disorder Examination-Questionnaire[9] *(EDE-Q) (baseline, mid-treatment, post-treatment, 6-month follow-up and monthly throughout treatment)* will be used to evaluate the frequency of binge eating episodes without the participant burden associated with the interview and shape and weight concerns. The EDE-Q has adequate psychometric properties. Only the three binge eating questions will be administered monthly. Participants respond to the following questions about binge eating; a) Over the past 28 days, how many times have you eaten what other people would regard as an unusually large amount of food? b) On how many of these times, did you have a sense of having lost control over your eating? c) Over the past 28 days, how many days have such overeating episodes occurred (i.e. you have eaten an unusually large amount of food and have a sense of loss of control at the time)? From these questions, number of binge eating episodes (eaten a large amount of food and felt a sense of loss of control) over the past 28 days and number of days participant experienced a binge eating episode can be calculated. We will also administer a modified question to identify monthly episodes of loss of control without large amount of food. a) Over the past 28 days, how many times did you have a sense of having lost control over your eating without having eaten an unusually large amount of food?

Anthropometry (baseline, mid-treatment, post-treatment, 6-month follow-up and all treatment visits): Height will be measured using a portable Schorr height board (Schorr Inc, Olney, MD) in triplicate. Height will be recorded to the nearest 0.1 cm for all trials, and the average of the 3 values will be used for analysis. Body weight in kilograms will be measured in duplicate on a Tanita Digital Scale (model WB-110A). Body weight will be recorded to the nearest 0.1 kg and the average of the 2 values will be used for analysis. Height and weight will be converted to body mass index (BMI=[kg/m2]). Waist circumference will be measured using a tape measure around the participant's middle around the waist and the measure recorded to the nearest cm will be recorded. The measurement will be taken 2-3 times and the average value maintained for analysis.

The Adult Eating Behaviour Questionnaire(AEBQ)[10] *(baseline, mid-treatment, post-treatment, 6-month follow-up and monthly during treatment)* consists of 35 questions, and 8 subscales, with responses on a 5-point scale ranging from "strongly disagree" to 'strongly agree". The *Satiety Responsiveness subscale* (SR) includes 4 questions and measures an individual's ability to respond to satiety signals (e.g., I often get full before my meal is finished). *The Food Responsivity subscale* (FR) includes 4 questions and measures an individual's reactivity to food cues in the environment (e.g., When I see or smell food that I like, it makes me want to eat).

Water Load Task (WLT-II)[11] (*baseline, post-treatment and 6-month post-treatment timepoints)* is a standardized, well tolerated and reliable method to assess gastric sensitivity. Participants drink room temperature, non-carbonated water ad libitum over two successive 5-min periods. During the first period, they are told to drink water until reaching the point of individually perceived fullness. Participants will be instructed to stop drinking when they feel the first signs of fullness. The volume of the water required to refill the flask to the initial level will be recorded. Then participants will be provided a second flask and told to drink again until reaching the point of maximum stomach fullness with directions. 'We now ask you to drink again during five minutes. Please continue drinking until your stomach is completely full, that is, entirely filled with water.' Participants are not told that there would be a second drinking phase in order not to influence their first water intake (i.e., to avoid that they would drink less in anticipation of the second drinking period). The flasks will be able to hold 5 liters of water but only filled to 1.5 liters to cap the amount of water that can be consumed.

Psychophysiological responding to food (baseline, post-treatment and 6-months post-treatment) is a measure of physical responses to highly craved foods. Electrophysiological recordings will all be sampled at 250 Hz. Participants complete three measurement phases in this task; baseline, food exposure, and recovery. During the baseline measurement phase and the recovery phase, participants will sit quietly for 6 minutes in a quiet private room. During the food exposure measurement phase, their highly craved food (identified by the participant) will be placed on the table in front of them. The participant is prompted to hold and smell the food and think about eating it for 6 minutes. Once the food exposure measurement phase is completed, the food is taken away and the participant completes the recovery phase (sit quietly for 6-minutes). Heart rate and Heart Rate Variability (HRV) will be measured using two Ag±AgCl electrodes, one attached on the left side of the subject, the other attached under the right collarbone. R-waves will be detected off-line with a template matching procedure, and inter-beat intervals will be calculated. Heart rate and HRV will be measured continuously during the food exposure tasks.

Reward-Based Eating[12] (all assessment time points and monthly during treatment). The Reward-Based Eating Drive scale (RED-13) was adapted from the RED-9 to evaluate a broader spectrum of reward-related eating. The RED-13 has demonstrated strong reliability and validity.

24-hour Dietary Recalls[13] (baseline, post-treatment and 6-month follow-up assessment time points). Dietary intake will be assessed with 3 24-hour dietary recalls on 3 non-consecutive days, via telephone interview outside of the assessment visit; two during the week and one on a weekend day. All interviews will use the Nutrition Data Systems for Research (NDS-R) nutrient calculation software. The multiple-pass interview technique will be used to prompt for complete food recall and descriptions and the Posner 2-dimensional food portion visual aid will be used to assist in portion size estimation. All dietary interviews will be administered by trained assessment staff from the UCSD Nutrition Shared Resource, blinded to study group assignment.

The Godin Leisure-Time Exercise Questionnaire[14] (GLTEQ; baseline, mid-treatment, post-treatment, 6-month follow-up and monthly during treatment) assesses the frequency and occurrence of leisure time physical activity. Participants report the number of times during a typical week that they engage in mild, moderate and strenuous exercise for more than 15 minutes.

Feasibility (throughout treatment). Feasibility will be assessed by Veteran attendance at treatment sessions.
Acceptability (post-treatment only). Acceptability will be assessed by survey, created by the study team based on established surveys used for Dr. Boutelle's previous NIH-funded studies (R01DK075861; R01DK094475). Likert-type ratings will be used to respond to questions, such as "How much did you enjoy the treatment?" and "How helpful did you find the treatment?"
Adherence (throughout treatment, post-treatment and 6-month follow-up). We will capture the amount of self-monitoring and the extent to which it is complete for all participants during treatment. Following treatment, we will ask participants to report to what extent they are following treatment recommendations.

Additional Food responsiveness tasks to food and non-food cues will be measured by the *stop signal task-food version*.[15,16] On each trial, participants are asked to discriminate between a picture of calorically-dense food or neutral object (e.g, chair). The two primary outcomes, stop signal reaction time for food pictures (SSRT-food) and neutral pictures (SSRT-neutral) will be compared to assess for food-specific impulsivity versus general impulsivity. Also, the
Dot-Probe[17] (attention bias to food) task will be used. During this task two stimuli (unhealthy food and other stimuli (either neutral object/healthy food)) are presented simultaneously side by side followed by a probe appearing in the location of one of the stimuli. Participants are instructed to respond as quickly as possible by pressing a corresponding key on a button box to indicate the location of the probe (similar to the dot probe task). Each trial starts with a central fixation cross, which remains on the screen for 100 ms. Subsequently, the target stimuli pair is presented for 200 ms or 2000 ms randomly. The probe is presented until the participant responds.

Measure	Instrument	Baseline	During TX	Mid TX	Post TX	6-mo F/U
**Anthropometry**	Height/Weight	X	X	X	X	X
	Waist Circumference	X		X	X	X
**Screening**	MINI[6]	X				
	Medical Conditions and Medications	X	X		X	X
	Demographics and Military info	X				
**Binge Eating**	EDE[7]	X		X	X	X
	EDEQ[9]	X	X		X	X
	BES[8]	X	X		X	X
**Satiety Responsiveness**	AEBQ[10]	X	X		X	X
	WLT-II[11]	X			X	X
	Intuitive Eating Scale[18]	X	X		X	X
**Food Responsiveness**	AEBQ[10]	X	X		X	X
	Psychophysiological Measures	X			X	X
	Power of Food Scale[19]	X	X		X	X

	Eating in the absence of hunger questionnaire[20]	X		X	X	X
	Stop-Signal Task[15, 16]	X			X	X
	Attention Bias Task[17]	X			X	X
	Food Craving Questionnaire[21]	X			X	X
	RED-13[12]	X		X	X	X
	Food Craving Acceptance and Action Questionnaire[27]	X		X	X	X
	Acceptance and Action Questionnaire for Weight-Related Difficulties (AAQ-W)[28]	X		X	X	X
**Mechanisms**	24 hour dietary recalls[13]	X			X	X
	Dietary History Questionnaire -III	X			X	X
	Godin Leisure[14]	X		X	X	X
**Moderators/Mediators**	UPPS-P[22]	X		X	X	X
	Perceived Stress Scale[23]	X		X	X	X
	BRIEF-A[24]	X		X	X	X
**Psychiatric**	PHQ-9[25]	X		X	X	X
	GAD-7[26]	X		X	X	X
**Feasibility**	Attendance		X			
**Acceptability**	Liking and helpfulness of treatment				X	
**Adherence**	Self-Monitoring		X			
	Adherence to treatment components				X	X

Quality control, fidelity and supervision of assessments. All assessors will be advanced graduate students, postdoctoral fellows or clinical psychologists who will attend a 2-day training with Dr. Eichen (study personnel) on the assessment methods for this study. All assessors will be videotaped and scored for adherence to the assessment procedures and certified by Dr. Eichen prior to working with study participants and will attend assessment supervision meetings with Dr. Eichen weekly. Assessment sessions will be audiotaped for ongoing performance monitoring. Random samples (e.g. 30% or more) of all interview sessions will be rated for fidelity by an independent rater using a measure created for this study and will be blind to treatment conditio n.

***Maximizing Retention*.** A systematic protocol will be followed to minimize subject attrition. Participants who miss a visit will be called by the group leader for counseling by telephone unless the missed visit was reported in advance (e.g., vacation). For each data collection visit, participants will be scheduled by telephone, sent written reminders, and called the day before the visit. Email addresses and phone numbers of the participants will be collected. Missed data collection visits will be rescheduled and followed up at least 3 times. If necessary, transportation to the clinic will be provided. If a participant withdraws from the study and does not wish to complete the full follow-up assessments, he or she will be given the option of having only his or her weight and binge eating assessed or self-reported in return for a $25 gift card at each assessment time point (mid-treatment, post-treatment, and follow-up) that remains after the point at which he or she withdrew.]
In order to ensure high retention of our sample, we will request personal e-mail addresses and cell phone numbers as well as contact information for two close friends or relatives to further enhance our ability to locate participants. In addition, after the first data collection is complete, we plan to send birthday, winter and summer holiday cards and newsletters in order to maintain updated addresses that will permit future co ntacts. Even if the participant moves multiple times before the follow-up survey administration, interim address information will be helpful in confirming location of an individual.

Audiorecording: All participants must agree to audiorecording of treatment and assessment sessions for fidelity, training, and supervision purposes. As this is mandatory, it is part of the regular consent process. The audiorecordings will not be shared with anybody outside of the research team or used for any other purpose so no additional audiorecording consent will be obtained. Recordings will be transferred to and kept on CHEAR's secure pediatric servers for up to 3 years after the study is completed or whenever fidelity rating is completed, whichever comes first.

Data Collection and Management: The data for this study will include a number of different methods of collecting data, including interviews, surveys and tasks. The survey will be available as a secure web-based survey. All surveys will be identified with the unique study ID rather than any identifying information. Following the first assessment visit, participants will be given the option of completing some surveys via the secure-web-based survey format prior to attending the in-person visit to reduce the amount of time they need to be present at CHEAR. If participants do not complete the surveys in advance, they will complete them as part of the visit. All surveys provided to be completed at home will include information that does not warrant immediate follow-up (i.e., no questions about suicidal ideation or self-harm). All surveys will be identified with the unique study ID rather than any identifying information. The psychophysiological data will need to be post-processed and entered and will be duplicate entered to minimize error. The data manager will evaluate range and means of all data collected and will identify data entry errors weekly. The data manager will create scales and will verify range and means. The data manager, along with Dr. Strong, will merge the data in preparation for analyses. These protocols have been successful in our other studies.

The data collected in this study once de-identified will be retained for future studies by UCSD or VA collaborators. These future studies will only have access to de-identified data. The only identifying information would include dates that participants attended treatment and assessments.

Analyses Plan

Primary Aim 1: Feasibility will be evaluated through the assessment of attendance and retention rates of individuals in the ROC and CBT treatments for treatment completers, dropouts and number of sessions attended. Logistic regression models will be used with planned covariates and baseline BMI to estimate between-group difference in odds of treatment completion, defined by receiving a sufficient dose of treatment (i.e. >70% of sessions). Cox proportional hazards models adjusted with planned covariates and baseline BMI to evaluate differences between groups in time to dropout defined as missing four consecutive treatment sessions. Descriptive data will be calculated and between-group differences in counts of sessions attended will be compared between groups using a generalized linear model to accommodate expected non-normal distributions and will include planned covariates and baseline BMI in the model. Model selection will proceed by testing assumptions and improvements in fit afforded by candidate models that could account for over-dispersion or a large number of participants with no-missed sessions (e.g. Poisson vs. negative binomial or zero-inflated counts). To assess acceptability, we will use linear models adjusted with planned covariates and baseline BMI to estimate between-group differences on the acceptability questionnaire, a continuous measure of both satisfaction and liking of the treatment they received.

Primary Aim 2: Efficacy of ROC and CBT on changes in binge eating *(as measured by the EDE; EDE-Q and BES)*, at mid-treatment, post-treatment and 6-months post-treatment timepoints. LME models with dummy-coded indicators for planned treatment comparisons between ROC and CBT, and a continuous term for time selected after evaluating non-linearity. Between-group (ROC vs. CBT) differences in change in binge eating (Primary Aim 2) will be estimated in models with planned covariates (age, gender), baseline BMI, and corresponding baseline values for each outcome. The GLME for counts of binge episodes and LME model for continuous symptom inventories provide maximum likelihood parameter estimates based on all of the available data, allowing for the inclusion of cases with missing data and the modeling of the covariance error structure of the data across the assessment points. Interactions of between-group indictor and time will assess whether differences in binge eating dissipate from active treatment compared to the 6-month follow-up timepoint.

Primary Aim 3: Efficacy of ROC and CBT on changes in weight loss (BMI) and energy intake (kcal/day), at mid-treatment, post-treatment and 6-months post-treatment time points will be evaluated with (G)LME models. Between-group (ROC vs. CBT) differences in change in BMI and energy intake will be estimated in models with planned covariates (age, gender), and corresponding baseline values for each outcome.

Secondary Aim 1: Efficacy of ROC and CBT on changes in food responsivity (FR scale; psychophysiological responding to food, reward-based eating) and satiety responsiveness (SR scale, WLT), using GLME adjusted for corresponding baseline values and planned covariates mirroring primary analyses. We also will use (G)LME to assess between-group differences in measures of reactivity to food cues using psychophysiological measures of EMG (salivation), heart rate and HRV at each follow-up.

Exploratory Aim 1: This aim will evaluate whether 1) individual factors including baseline age, gender, binge eating status, satiety responsiveness, responsivity to food cues, or reward-based eating moderate the impact of ROC vs CBT on improvements in binge eating and weight by evaluating two interaction terms in primary outcome models, and whether 2) improvements in responsivity to food cues, satiety responsiveness, reward-based eating or physical activity mediate improvements in binge eating and weight, by screening each in single mediator models prior to estimating joint effects food cue responsiveness, satiety responsiveness, or reward-based eating mediate improvements in binge eating, using a multiple mediator model. Significance tests of indirect effects will be assessed using a product of coefficients method with bootstrap estimation of 95% confidence intervals.

10. HUMAN SUBJECTS

Overall, we expect to consent 300 individuals to randomize and have at least 120 Veterans and civilians (if needed) start treatment who meet the following eligibility criteria:

Enrollment criteria:

1. All participants will be Veteran or civilian men and women between the ages of 18-65 meeting criteria for overweight, with a BMI $\geq$25.
2. Participants will meet Diagnostic and Statistical Manual–5 (DSM-5) criteria for BED or subclinical BED
3. Participants will be willing and able to participate in assessment visits and treatment sessions at University of California, San Diego (UCSD).
4. Participants will be able to provide data in English through interviews and questionnaires and understand treatment materials in English.
5. Participants will be willing to maintain contact with the investigators for 11 months.
6. Participants will be free of serious or unstable medical (current symptoms of angina, stroke, heart disease or other serious medical condition that would make physical activity unsafe or impossible at a moderate level) or psychiatric illness (i.e., active suicidal ideation, history of suicide attempt within 1 year, current unmanaged psychosis, manic episode, anorexia nervosa, bulimia nervosa, or substance abuse within the past year) or psychosocial instability (e.g., homelessness) that could compromise study participation.
7. Participants will be free from conditions in which exercise or weight loss will be detrimental to the participant's health (e.g., pregnancy); pharmacotherapy for obesity or binge eating disorder (e.g., Orlistat or Meridia) or bariatric surgery within the past 6 months or planning to start such treatments in the next 11 months.
8. Participants will not be moving out of the San Diego area for the duration of their study enrollment (11 months).
9. Participants will not be pregnant, planning to get pregnant in the 11-month study period or lactating.
10. Participants will not be taking medication that may impair physical activity tolerance or performance (e.g., beta blockers)
11. Participants will not be participating in group or individual psychotherapy for binge eating or weight management.
12. Participants with medical or psychological problems or taking medications that could make adherence with the study protocol difficult or dangerous will not be included.

11. RECRUITMENT AND PROCEDURES PREPARATORY TO RESEARCH

Participants will be recruited from the San Diego Metropolitan community and the VA San Diego Healthcare System (VASDHS). Dr. Afari (co-investigator) and her team will recruit Veterans across multiple clinics including weight control, primary care, and mental health clinics. In addition to recruitment through clinics, Dr. Afari will recruit Veterans through identifying potential participants through medical records, physician referrals, and electronic and paper flyers. Dr. Afari and her team will consent potential participants using the VA consent form which covers these pre-screen activities and will pre-screen Veterans from VASDHS for initial potential eligibility and interest. The brief pre-screening will help screen for inclusion/exclusion criteria and be similar to the online/phone screen used at UCSD.

Upon initial eligibility and interest, the VASDHS team will invite eligible participants to attend an in-person orientation at UCSD and provide the UCSD research team the contact information those who are eligible and UCSD staff will complete all remaining study procedures starting with orientation and UCSD consent. The recruitment efforts of the VA staff will be covered under the VASDHS IRB and participants recruited through this method will be the only participants who sign the VA consent form which will cover Dr. Afari and her staff obtaining the information needed to prescreen participants.

Additional efforts will be made to recruit Veterans from the community as well to capture individuals who may not receive their healthcare at the VA and maximize efforts to recruit Veterans for this study. The Center for Healthy Eating and Activity Research (CHEAR) at UCSD recruits through newspaper ads, online advertisements, social media, radio ads, online marketing and physician referrals. In an effort to speed recruitment, CHEAR hired Bump in 2017, a company that provides online recruitment campaigns and software. Bump uses proprietary algorithms developed through machine learning to target profiles for specific participant groups. Bump may advertise on Facebook, Google, Instagram, medical informational sites, and other relevant networks. Every effort will be made to recruit only Veterans for this study. However, should any significant recruitment delays occur, the team will consider the recruitment of active duty service members in the community and civilians if necessary who meet inclusion/exclusion criteria to assure recruitment milestones are met in a timely fashion to complete the trial and have sufficient power.

Veterans who do not complete the prescreening with VASDHS staff, will be asked to complete a brief (~10 minute) secure online survey to screen for inclusion/exclusion criteria (age, self-reported binge eating, BMI, medication exclusions, psychiatric exclusions, pregnancy). If any participants do not wish to complete this online, they could complete it on the phone with a CHEAR staff member along with the phone screen. Those who remain eligible after the online screen, will complete a phone screen with a trained CHEAR staff member to further assess inclusion and exclusion criteria.

All potential participants who remain eligible after the prescreening by VASDHS staff or CHEAR staff will attend a brief orientation at CHEAR, conducted by Dr. Boutelle or CHEAR study staff, which will describe the study, the two treatments arms, randomization methods, expectations for participation, and incentives for time and effort. Following the orientation, those who remain interested, will undergo the UCSD consent process and will sign consent forms, have their height and weight measured, and be scheduled for their first assessment visit. This recruitment and orientation process is used in Dr. Boutelle's current and past NIH funded studies and serves to educate potential research participants on randomized controlled trial research process, the current study, and the informed consent process.

12. INFORMED CONSENT

Participants recruited at the VA will sign the VA consent form (covered under the VA IRB) to cover initial screening.

For participants recruited directly through UCSD, a waiver of documented consent is requested for the online screening and phone screening process (UCSD recruited participants only). Prior to baseline visit, the online screening will assess for basic inclusion and exclusionary criteria. This process presents no more than minimal risk of harm to subjects, and involves no procedures, for which written consent is normally required outside of the research context. For individuals who do not have online access or wish to provide the information online, the questions asked on the online screen can be administered over the phone in conjunction with the phone screen. The phone screening will assess for basic inclusion and exclusionary criteria. This process presents no more than minimal risk of harm to participants, and involves no procedures for which written consent is normally required outside of the research context. For these reasons, we request a waiver of documented consent for the phone screen.

All participants, regardless of how they were recruited will complete UCSD written informed consent prior to enrollment at an orientation session. It is not likely that participants for this population will lack the capacity needed for consent. If there are any concerns, to ensure participants understand the consent form, they will be asked to describe what the study is about. For participants in which capacity for consent is judged to be questionable, they will complete the Mini-mental State Examination (MMSE) and if they receive under a score of 24, they will not be

eligible for enrollment (i.e., surrogate consent will not be acceptable). It is highly unlikely that the MMSE will need to be administered to this patient population but if it is administered, documentation of the test results will be kept in the participant file.

The orientation and UCSD consent process may take place with a group of potential participants. Participants may ask questions as a group and they will also be informed that they can each have an opportunity to privately ask additional questions. If individuals are unsure as to whether they are willing to participate in the study, they may take the consent form home with them. If they later decide they wish to participate, they may schedule their first assessment and sign the consent form prior to participating in the assessment.

CHEAR staff members able to provide information about the study and carry out the consent procedures include Kerri Boutelle, Dawn Eichen, Cyrielle Hatcher, Zoe Mestre, Sara Appleton-Knapp, Takisha Corbett, Michael Manzano, Natalie Alamo, Kaylen Moline and Saori Obayashi.

13. ALTERNATIVES TO STUDY PARTICIPATION

The alternatives to participation in this study are to not participate and to seek treatment with another therapist or community program.

14. POTENTIAL RISKS

1. Degree of inconvenience for Veterans who may miss work, activities, meetings, etc. to participate in the study
2. Potential risk of psychological assessments. For some participants, disclosing potential information about mental health symptoms and eating behaviors may be uncomfortable. Questions regarding individual behaviors, emotions or attitudes may be considered sensitive to some participants.
3. Potential risk of psychological treatment. As in all therapies, treatment for binge eating and weight may encourage discussion of sensitive or distressing topics which may provoke emotional responses for some participants. The ROC treatment involves exposure to food while restricting consumption of said food. This may provoke negative emotions for some participants. Further, participants in some treatment arms will be encouraged to increase their level of physical activity. Improper or too-vigorous exercise or physical activity, if performed by the participant against the advice of the research staff, could be associated with injury or discomfort.
4. Potential risk of loss of confidentiality: Risk associated with breach of confidentiality of behavioral research data. Since this study includes psychological assessments as well as height and weight, there is the potential that this information might not be kept confidential (for instance by theft of study material).

15. RISK MANAGEMENT PROCEDURES AND ADEQUACY OF RESOURCES

There is a degree of inconvenience for Veterans who may miss work, activities, meetings, etc. to participate in the study. It is possible that missed work could result in decreased income. At CHEAR, assessments are conducted in the morning, afternoon, evening and on Saturdays if necessary to reduce the impact of assessments on Veteran's work or activities. Participants will be made aware of the weekly treatment night during the screening process. If a Veteran is busy with activities or other commitments on that evening, they will be offered to wait for another cohort which will be offered on an alternative evening.

There is a risk of reactivity to assessment procedures. For some participants, disclosing information about mental health symptoms and eating behaviors may be uncomfortable. Questions regarding individual behaviors, emotions or attitudes may be considered sensitive to some participants. This risk will be mitigated by having well supervised assessors conduct the assessments, thereby being aware of emotional reactivity or stress response in an early form. Veterans will be notified that they may decline to answer any questions due to discomfort or may choose to end their participation in screening if no longer interested.

Risk management for psychiatric concerns identified in assessment

For any unidentified/unreported psychiatric concerns identified during assessments for this project, we will execute the following protocol:

- The Veteran will be notified of concerns identified.
- The Veteran will be given a list of referrals in the community or referred back to the VASDHS mental health team (depending on source of recruitment). Dr. Boutelle and Dr. Bernard are both licensed clinical psychologists, and experienced in assessing psychiatric risk. CHEAR has an extensive list of community mental health referrals, some which offer treatment at no or low-cost.
- If significant concern is warranted (i.e. active suicidal ideation, risk of intent to hurt self or others), participants will be immediately assessed by a licensed clinical psychologist at CHEAR (of which there are 5 at this time), and an appropriate psychiatric referral will be made. Should any imminent concerns be present and a participant's safety cannot be guaranteed, Dr. Boutelle or another licensed psychologist at CHEAR will call the Psychiatric Emergency Response Team (PERT). PERT consists of specially trained officers and deputies who are paired with licensed mental health professionals. Together, they respond on-scene to situations involving people who are experiencing a mental health related crisis and can initiate involuntary hospitalization and provide crisis management as necessary.

There is a risk of reactivity to treatment procedures. As in all therapies, treatment for binge eating and weight may encourage discussion of sensitive or distressing topics which may provoke emotional responses for some participants. This risk will be mitigated by having well supervised interventionists conduct the treatments, thereby being aware of emotional reactivity or stress response in an early form, and conducting treatment in such a way as to reduce such responses. Should a participant become symptomatic during treatment, the interventionist will assess the problem, and in consultation with Dr. Boutelle and Dr. Bernard, decide whether to withdraw the individual from treatment and/or to refer them for treatment elsewhere. Participants will be encouraged to talk to their group leaders, study coordinator or the principal investigator if they experience distress in treatment. Participants will be reminded they can withdraw their participation at any time without consequence. If any staff member at CHEAR has concerns that participation in treatment is negatively affecting a participant, the staff member will discuss the case with Dr. Boutelle and/or Dr. Bernard who can meet with the participant and make the decision to withdraw participation of any participant from the study without their consent if it is in their clinical best interest. All participants who are withdrawn will be provided referrals in the community and/or referred back to their mental health provider.

There is a risk of injury. In the ROC arm, Veterans will be encouraged to increase their physical activity. Although research staff will help tailor the physical activity recommendations to a Veteran's physical or medical status, it is possible that improper or vigorous physical activity could be associated with injury or discomfort. In order to address this risk, Veterans will self-report orthopedic problems, gait disturbances, and medical status prior to enrolling in the study. All participants will be instructed to alert the research team of any injury sustained as soon as possible. If an injury has occurred, we will request that the participant follow-up with his/her primary care provider before continuing with the physical activity recommendations in the study.

Risk of loss of confidentiality as a direct result of participation in the recruitment, screening, assessment and treatment. There is a very small risk that identified data may be compromised as part of this study. All data collected during the study assessments or treatment is de-identified and only labeled with an ID number, no name or other identifying information. With regards to paper forms, consent forms with identifying information are stored in a locked room in a locked filing cabinet. Hard copies of de-identified data is stored in a separate locked room in a locked filing cabinet. Any electronic document with identifiable information is password protected and the password is only provided to essential study staff who need to access that document to aid in the conduct of the study. Electronic de-identified data is stored on encrypted firewall protected UCSD servers or on REDCAP and only accessible by study staff. Participants will not be identified in any reports or publications. Appropriate parties other than the UCSD research staff may be granted access to the study data as required (e.g., the sponsor of this study, the Department of Defense, UCSD IRB personnel, medical monitors, or other similar entities); these individuals will be bound by the same rules of confidentiality as the investigative team not to reveal the participant's identity to others. The only time confidentiality will purposefully be broken is when required by law if the study team receives information that a participant is a threat to him/herself, another specified target, or regarding actual or reasonably suspicious child, dependent adult, or elderly abuse or neglect.

There is a risk that a Veteran may know another Veteran in the waiting room or treatment group. We have experience managing this kind of loss of confidentiality. Both participants will be reminded of the confidentiality

expectations in a treatment study, and will meet with trained CHEAR staff individually to assess participant concerns. This method has been successful to date in our other studies, and most times, participants are not concerned about individual they know. If a Veteran has concerns about a familiar individual, the Veteran with the concern will be offered an option to participate in a future cohort.

For the transfer of screening data from the VASDHS staff to the UCSD CHEAR recruitment staff, a separate IRB will be filed under the VA to cover the consent and HIPAA exceptions of the pre-screening process of Veterans at the VA and any information used to determine eligibility. These forms will give permission for Dr. Afari's team to share the names and screening data with UCSD CHEAR study staff. Dr. Afari's team will provide copies of the screening data either on a password-protected document electronically or on a password protected cd with names and contact information stored separately linked to an ID. Only the VASDHS staff and the CHEAR recruitment staff will have the password to unlock the CD or files. The VA will securely store the electronic data according to VASDHS IRB and policies as well as securely keep the consent forms in locked filing cabinets in which only the VASDHS study team will have access to them.

To help maintain confidentiality of participants, if emailing more than one participant (e.g., emailing about reminder for treatment session), all participant's email addresses will be kept confidential by using the BCC function and no participant names will be included. When emailing participants from email templates, staff will copy from our templates instead of forwarding from emails sent to other participants and to always start new e-mail documents to ensure no participant email information is accidentally disclosed to another participant.

Data Safety and Monitoring (DSM): Because of this low risk status, the data and safety monitoring plan (DSMP) for this trial focuses on close monitoring by the principal investigator (PI) in conjunction with a safety officer, along with prompt reporting of excessive adverse events and any serious adverse events to the DoD and to the IRB at the University of California San Diego. The safety officer for this trial will be Scott Crow, MD. Dr. Crow is a psychiatrist with over 30 years of experience in the assessment and treatment of patients with eating disorders and obesity in research trials and in clinics. Dr. Crow is a Professor at the University of Minnesota, Psychiatry Department and the Medical Director of the Emily Program in Minnesota. As a Safety Officer, Dr. Crow will review the reports sent by the study coordinator (at the frequency outlined below) and will use a checklist to determine whether there is any corrective action, trigger of an ad hoc review, or stopping rule violation that should be communicated to the study investigator, the University of California San Diego IRB, and the funding agency.

Safety reports will be sent to Dr. Strong (statistician), Dr. Boutelle (Principal Investigator), and the safety officer (Dr. Crow). The Project Coordinator will be responsible for assembling the data and producing these reports, as well as assuring that all parties obtain copies of these reports.

The frequency of data review for this study differs according to the type of data and can be summarized in the following table:

Data type	Frequency of review
Subject accrual (adherence to protocol regarding demographics, inclusion/exclusion)	At the end of each recruitment wave
Adverse event rates (injuries)	Quarterly
Stopping rules report regarding statistical power implications of drop outs and missing data	Yearly

Stopping rules: In this minimal risk intervention trial, it is more likely that drop-outs or difficulty in recruiting adequate numbers of participants will require stopping the trial, than that of excess adverse events will occur and require stopping the trial. However, as outlined elsewhere, we will monitor injury rates in all participants and the safety officer, together with the study investigators, will alert the IRB and the DOD if a larger than reasonably expected injury rate should occur in the treatment group.

Measurement and reporting of adverse events. Serious adverse events will be those that result in death, are life threatening, require (or prolong) hospitalization, cause persistent or significant disability/incapacity, result in congenital anomalies or birth defects, or are other conditions which, in the judgment of the investigators, represent significant hazards. Serious adverse events will be reported by the PI verbally and in writing as soon as possible to the IRB and

as appropriate to the DoD. Adverse events will be those that include any unfavorable and unintended diagnosis, symptom, sign, syndrome or disease which either occurs during the study, having been absent at baseline, or, if present at baseline, appears to have worsened. All adverse events (serious and non-serious, expected and unexpected) will be reported to the UCSD IRB at timelines according to appropriate guidelines (e.g., expected and not study related at IRB continuing review; unanticipated problems involving risk to participants within 10 working days) and reported to the study monitor quarterly if anticipated and not study related, or within the same timeline as reporting to the IRB if sooner than quarterly. All unanticipated problems involving risk to subjects or others will be promptly reported to the UCSD IRB and the DoD.

Blinded adverse events data will be presented to the study statistician, the PI, and the safety officer throughout this trial. The adverse event log will be used by the study staff to report injuries that may be associated with this intervention. Very few injuries are expected, as the main focus of treatment is changing eating behavior. The physical activity recommendations will include both vigorous and lifestyle physical activities, which can be adapted to the Veteran's physical or medical status.

It is not likely but should any adverse events occur during screening at the VASDHS they will submit those to the VASDHS IRB and follow all protocols and report to the DoD and DSMP as appropriate.

16. PRIVACY AND CONFIDENTIALITY CONSIDERATIONS INCLUDING DATA ACCESS AND MANAGEMENT

We will implement the following security plan to promote security of the data and privacy of the participants. Data collection will be completed with an emphasis upon maintenance of confidentiality. We plan to extract data from questionnaires and in-study behavioral measures. Only Dr. Boutelle and her research staff, each of whom has mental health clinical training and UCSD clearance will have access to any personal health information collected. We will assign participants a study identification number unrelated to identifying information. The study ID number will be used by participants on their questionnaires and data collection forms. The only materials containing subject identifying information will be the consent and HIPAA forms. We will create a master list linking the de-identified study identification number to the participant's record. The record in the master list will be identified by a randomized participant number provided by our statistician. The master list will be maintained by Dr. Boutelle in her laboratory. There will be only one password protected electronic version of this file. Access to the master list will be limited to the P.I.s and their designees, all of whom will have completed DoD and UCSD IRB training requirements. At the earliest opportunity and no later than 36 months following data analysis, the master list (i.e., the only source that links the study identification numbers to the individuals) will be destroyed.

The online screen will be set up through Research Electronic Data Capture (REDCap). REDCap is a secure web-based application built around HIPAA guidelines to support data capture for research studies. UCSD's Clinical and Translational Research Institute (CTRI) provides support to facilitating conducting research through REDCap as well as comprehensive maintenance and support. The REDCap Software sits on a dedicated server behind a firewall to eliminate all non-essential credentials. The data itself is isolated on a separate server that sits behind an additional firewall layer for added security. Interaction between REDCap and the participant is encrypted via SSL. support for the REDCap infrastructure at UCSD. Only essential staff (Data Manager, Project Coordinator, RAs) can access the data collected through REDCap through log-in credentials granted though UCSD's Business Systems Single Sign-On. De-identified data collected during treatment or in person assessments will also be entered through REDCap using the participant's ID number so only approved study staff can access the database.

Screening information received from the VASDHS will be securely stored and password protected on our UCSD servers with only study staff having access to this data.

17. POTENTIAL BENEFITS

The most important potential benefit is the reduction of binge eating, which may translate into a number of favorable correlated outcomes, including improved weight loss, weight loss maintenance, and reductions in psychological distress.

18. RISK/BENEFIT RATIO

There is a relatively low risk to participants and the potential to benefit from a reduction in binge eating and weight.

19. EXPENSE TO PARTICIPANT

There is no expense to participants for participating in this study. There is no cost to participate.

20. COMPENSATION FOR PARTICIPATION

All participants will receive incentives at the following levels for time and effort: baseline = $50, mid-treatment = $50, post-treatment = $100, 6-month follow-up = $150. Participants who withdraw, but who wish to have their weight and binge eating measured or self-report their weight and binge eating to study staff at follow-up time points will receive a $25 gift card at each assessment time point (mid-treatment, post-treatment, mid-follow-up, and follow-up) that remains after the point at which he or she withdrew. Participants who enroll and complete all assessment visits can earn up to $350.

21. PRIVILEGES/CERTIFICATIONS/LICENSES AND RESEARCH TEAM RESPONSIBILITIES

Kerri Boutelle, Ph.D (PI) is a professor at UCSD in the Departments of Pediatrics, Family Medicine and Public Heath, and Psychiatry and is a licensed clinical psychologist. Her research specializes in the study of obesity and eating disorder behaviors. Dr. Boutelle is also a key developer of the ROC program for binge eating and weight being tested in this proposed study and has unique expertise in the application of this treatment.

David Strong, Ph.D (Co-I) is a professor in the department of family medicine and a licensed clinical psychologist. He has extensive experience managing data and conducting data analyses. His responsibilities include managing the databases and conducting data analyses for this project.

Dawn Eichen Ph.D. is a postdoctoral fellow and a licensed clinical psychologist at UCSD. She will be responsible for training, supervising and certifying assessors. She will also conduct assessments and treatment.

Takisha Corbett PhD. is a postdoctoral fellow at UCSD. She will assist with project management as well as assessment and intervention.

Saori Obayashi Ph.D. is a licensed dietician and a Staff Research Associate at UCSD. She will assist with project management as well as assessment and intervention.

Eastern Kang, Ph.D. is a postdoctoral fellow at UCSD. He will oversee data analyses including evaluating data entry accuracy, merging of data files, creation of scales, and evaluating initial frequencies and means.

Sara Appleton-Knapp Ph.D. is a project scientist at UCSD. She will assist with project management as well as assessment and intervention.

Rebecca Bernard is a licensed clinical psychologist at UCSD. She will supervise the interventionist in the study and will assist with assessment and intervention.

Kaylen Moline is the recruitment coordinator for CHEAR at UCSD. She will coordinate all the recruitment and maintenance of the cohort for the study.

Michael Manzano is a graduate student at CHEAR at UCSD. He will assist with project management as well as assessment and intervention.

Zoe Mestre is a graduate student at CHEAR at UCSD. She will assist with project management as well as assessment and intervention.

Natalie Alamo is a Staff Research Associate at UCSD. She will assist with project management as well as assessment and intervention.

The following individuals are part of the CHEAR Assessment staff. They include graduate students and recent graduate program graduates who conduct clinical assessments and can serve as group leaders or co-leaders. They may also introduce the study and obtain consent. Everyone has CITI training:
Natalie Alamo
Alexis Alvarez
Daylin Anderson
Lia Bauman
Aliya Dincer
Monica Dixon
Nicole Goren
Cyrielle Hacher
Stephanie Kessl
Kelsie Kinden
Zoe Mestre
Carolina Gonzalez
Sarah Speers

The following individuals are Research Assistants at CHEAR. These include undergraduate research assistants and high school volunteers who help oversee the assessments and introduce the studies to the participants and may obtain consent. They also may help assist with group material preparation and obtaining heights/weights. Everyone has verified CITI training prior to beginning.
Research assistants:
Paige Adam
Jade Baltierra
Angela Camodeca
Karina Collins
Sophie Chamaa
Kamilah-Andrea Cunanan
Jemima Degamo
Madison Doty
Cailey Gembowski
In “Ashley” Haong
Nawal Helo
Marika Kobayashi
Aiko Kumano
Kayla Larot
Tiffany Luu
Victoria Mac
Sierra Marie
Michelle Ng
Isabela Pascua
Samantha Pecenka
Yasmine Saraf
Heather Spaun
Allison Tegner
Grace Tran
Sharon Tu
Katrina Waters

VASDHS Collaborators
Niloo Afari, Ph.D. (Co-I) is Research Scientist at the Veterans Medical Research Foundation and a Staff Psychologist/ Associate Chief of Staff for mental Health at VASDHS. She is a Co-investigator on the grant and the site-PI at the

VASDHS. She will oversee the overall conduct of the study recruitment at the VASDHS and communicate with the UCSD team to ensure collaboration. Dr. Afari will also work closely with the overall study team to provide input on study design, data collection and analysis, and assisting with publications and presentations.

22. BIBLIOGRAPHY

1. Yager J, Devlin M, Halmi K, Herzog D, Mitchell J, Powers D, Zerbe K. Guideline Watch (August 2012): Practice Guideline for the Treatment of Patients With Eating Disorders. 3rd ed. American Psychiatryc Association. 2012:1-18.
2. Brownley KA, Berkman ND, Peat C, Lohr K, Cullen K, Bann C, Bulik C. Binge-eating disorder in adults: A systematic review and meta-analysis. Ann Intern Med. 2016;165(6):409-20. doi: 10.7326/M15-2455; PMID: 27367316.
3. Grilo C, Masheb R, Wilson G, Gueorguieva R, White M. Cognitive-behavioral therapy, behavioral weight loss, and sequential treatment for obese patients with binge-eating disorder: a randomized controlled trial. J Consult Clin Psychol. 2011;79(5):675-85. doi: 10.1037/a0025049; PMID: 21859185. PMCID: PMC3258572.
4. Schachter S. Some extraordinary facts about obese humans and rats. Am Psychol. 1971;26(2):129-44; PMCID: 5541215.
5. Boutelle N, Knatz S, Carlson J, Bergmann K, Peterson C. An open trial targeting food cue reactivity and satiety sensitivity in overweight and obese binge eaters. Cogn Behav Pract. 2017;24(3):363-73. doi: https://doi.org/10.1016/j.cbpra.2016.08.003.
6. Sheehan DV, Lecrubier Y, Sheehan KH, Amorim P, Janavs J, Weiller E, Hergueta T, Baker R, Dunbar GC. The Mini-International Neuropspychiatric Interview (M.I.N.I.): the development and validation of a structured diagnostic psychiatric interview for DSM-IV and ICD-10. J Clin Psychiatry. 1998;59(20):22-33;quiz 4-57; PMID: 9881538.
7. Cooper Z, Fairburn C. The eating disorder examination: semi-structured interview for the asessment of the specific psychopathology of eating disorders. Int J Eat Disord. 1987;6:1-8. doi: 10.1002/1098-108X(198701)6:1<1::AID-EAT2260060102>3.0.CO;2-9.
8. Gormally J, Black S, Daston S, Rardin D. The assessment of binge eating severity among obese persons. Addict Behav. 1982;7(1):47-55.
9. Fairburn C, Beglin S. Eating Disorder Examination Questionnaire (EDE-Q 6.0) C.G. Fairburn (Ed.), Cognitive behavior therapy and eating disorders. Guilford Press, New York. 2008:309-13.
10. Mallan KM, Fildes A, de la Piedad Garcia X, Drzezdzon J, Sampson M, Llewellyn C. Appetitive traits associated with higher and lower body mass index: evaluating the validity of the adult eating behaviour questionnaire in an Australian sample. Int J Behav Nutr Phys Act. 2017;14(1):130. doi: 10.1186/s12966-017-0587-7; PMID: 28938904. PMCID: PMC5610469.
11. van Dyke Z, Vogele C, Blechert J, Lutz A, Schulz A, Herbert B. The Water Load Test as a measure of gastric interoception: development of a two-stage protocol and application to a healthy female population. Plos One. 2016;11(9):e0163574. doi: 10.1371/journal.pone.0163574; PMID: 27657528.
12. Mason A, Vainik U, Acree M, Tomiyama A, Dagher A, Epel E, Hecht F. Improving Assessment of the Spectrum of Reward-Related Eating: The RED-13. Front Psychol. 2017;8:795. doi: 10.3389/fpsyg.2017.00795; PMID: 28611698.
13. Galasso R, Panico S, Celentano E, Del Pezzo M. Relative validity of multiple telephone versus face-to-face 24-hour dietary recalls. Ann Epidemiol. 1994;4(4):332-6; PMID: 7921324.
14. Godin G, Shephard R. A simple method to assess exercise behavior in the community. Can J Appl Sport Sci. 1985;10(3):141-6; PMID: 4053261.
15. Verbruggen F, Logan G, Stevens M. STOP-IT: Windows executable software for the stop-signal paradigm. Behav Res Methods. 2008;40(2):479-483.
16. Nederkoorn C, Coelho JS, Guerrieri R, Houben K, Jansen A. Specificity of the failure to inhibit responses in overweight children. Appetite. Oct 2012;59(2):409-413.
17. Beard C, Sawyer AT, Hofman SG. Efficacy of Attention Bias Modification Using Threat and Appetitive Stimuli: A Meta-Analytic Review. *Behavior Therapy.* 2012;43(4):724-740.
18. Tylka TL, Kroon Van Diest AM. The Intuitive Eating Scale – 2: Item Refinement and Psychometric Evaluation with College Women and Men. *Journal of Counseling Psychology*. 2013;60(1):137-153.

19. Lowe MR, Butryn ML, Didie ER, et al. The Power of Food Scale. A new measure of the psychological influence of the food environment. *Appetite*. Aug 2009;53(1):114-118.
20. Tanofsky-Kraff, M. et al. (2008). Psychometric properties of a new questionnaire to assess eating in the absence of hunger in children and adolescents. Appetite, 51:148-155.
21. Cepeda-Benito, A., Gleaves, D. H., Williams, T. L., and Erath, S. A. The development and validation of the state and trait Food-Cravings Questionnaires. Behav. Ther. 2000;31:151–173. doi: 10.1016/S0005-7894(00)80009-X
22. Whiteside SP, Lynam DR, Miller JD and Reynolds SK. Validation of the UPPS impulsive behaviour scale: A four-factor model of impulsivity. *Eur. J. Pers*. 2005;19:559–574
23. Cohen S, Kamarck T, and Mermelstein R. A global measure of perceived stress. *Journal of Health and Social Behavior*. 1983;24:386-396.
24. Roth, R.M., Isquith, P.K., Gioia, G.A. Behavior rating inventory of executive function—Adult version: Professional manual. Psychological Assessment Resources, Lutz, FL. (2005).
25. Kroenke K, Spitzer R, Williams W. The PHQ-9: Validity of a brief depression severity measure. Journal of General Internal Medicine. 2001;16:606-616.
26. Spitzer RL, Kroenke K, Williams JB, & Lowe B. A brief measure for assessing generalized anxiety disorder: the GAD-7. 2006; 166:1092-1097
27. Lillis, J., & Hayes, S.C. (2008). Measuring avoidance and inflexibility in weight related problems. International Journal of Behavioral Consultation and Therapy, 4(4), 348-354.
28. Juarascio, A., Forman, E., Timko, C. A., Butryn, M., & Goodwin, C. (2011). The development and validation of the food craving acceptance and action questionnaire (FAAQ). Eating behaviors, 12(3), 182-187.

23. FUNDING SUPPORT FOR THIS STUDY

Funding for this study will be provided by the Department of Defense award number PR170320

24. BIOLOGICAL MATERIALS TRANSFER AGREEMENT

Not applicable. No biological materials will be collected or transferred.

25. INVESTIGATIONAL DRUG FACT SHEET AND IND/IDE HOLDER

Not applicable. No drugs will be given or investigated in this study.

26. IMPACT ON STAFF

Not applicable. This study does not involve the nursing staff from UCSD and/or VASDHS.

27. CONFLICT OF INTEREST

There are no conflicts of interest.

28. SUPPLEMENTAL INSTRUCTIONS FOR CANCER-RELATED STUDIES

Not applicable.

29. OTHER APPROVALS/REGULATED MATERIALS

None.

30. PROCEDURES FOR SURROGATE CONSENT AND/OR DECISIONAL CAPACITY ASSESSMENT

Not applicable. Surrogate consent will not be used for this study.

Modifications to the Protocol (excluding staff and advertising changes)

Initial approval July 2018

January 2019

- Screening: Added a question about whether the participant received healthcare at VA San Diego Healthcare System (VASDHS) to online screen. Added permission to share information between VASDHS and UC, San Diego.
- Assessment visits: Increased baseline, post-treatment, and 6-month follow-up assessments from one visit to two visits to collect additional measures
- Assessment measures: Added the following to the demographic questionnaire: first language and primary language. Added the following measures to baseline, post-treatment and 6-month follow-up: Yale Food Addiction Scale, PTSD Checklist for DSM 5, Wide Range Achievement Test, Delayed Discounting, Matrix Reasoning Test, and Digit Symbol Coding
- Assessment measures: Changed from one mid-treatment assessment to monthly assessments for the following measures: Godin Leisure-Time Exercise, Eating Disorder Examination Questionnaire, Binge Eating Scale, Adult Eating Behavior Questionnaire, Reward Based Eating Drive Scale-13.
- Treatment: Increased the length of treatment sessions from 60 minutes to 90 minutes
- Inclusion/exclusion criteria: Changed the enrollment criteria from BMI>25 to BMI $\geq$ 25. Specified exclusion of individuals with Bipolar and Borderline Personality Disorder.
- Incentives: Increased and reallocated incentives in accordance with the increased number of assessments: Total of $415 consisting of $25 x 2 baseline assessment visits, $5 to cover transportation x 18 treatments=$90, $25 for one mid-treatment assessment visit, $100 for two post-treatment assessment visits, and $150 for two 6-month follow-up assessment visits. Added scrips (check) as an alternative incentive to gift cards.

March 2019

- Assessment visits: Changed randomization visit from in-person to over the phone
- Assessment measures: Added additional category for gender and race (other [please explain]), replaced primary language with preferred language, added additional category for the branch of the military, replaced rank in the military with exposure to combat, and modified income questions in the demographic questionnaire.
- Assessment measures: Added the following measures to baseline, post-treatment and 6-month follow-up: digit span, eye tracking, and Global Physical Activity Questionnaire (GPAQ). Added questions regarding weight management practices prior to participating in the study.
- Inclusion/exclusion criteria: Removed the exclusionary criteria “taking medication that may interfere physical activity”.

May 2019

- Assessment measures: Added a question requesting permission to communicate with physician for safety concerns or collecting weight information.

October 2019

- Screening: Changed from online screen to phone screen question whether they receive health care at VASDHS.

March 2020

- Requested permission from IRB to share study participants' data with VA Boston Healthcare System for another DOD funded study.

April 2020

- Screening: Added a question to determine if other family members are participating in the current study to phone screen.
- Assessment measures: Added the following questions to the demographic questionnaire: sex assigned at birth, gender identify, years served as active-duty personnel vs. reservist, branch served, positions served as active-duty personnel and when a reservist, highest rank at discharge, number of years since served in the military, whether on reserve while participating in the study.
- Recruitment: Added permission for current/former participants to share information about the study with friends and family.
- Consent: Added teleconsent, virtual assessments, and virtual treatments via password protected HIPAA compliant Zoom meetings in response to COVID-19 pandemic restrictions.
- Incentives: Added direct deposit as an incentive option to gift cards and scrips.

April 2021

- Recruitment: Added the use of BuildClinical for recruitment.

May 2021

- Increased the number of potential participants who could consent from 300 to 350 (to randomize 120 participants) since conversion rate was lower than expected.
- Incentives: Removed incentive for travel as it was no longer needed once sessions were remote.

June 2021

- Screening: Added 3 questions regarding loss of control eating to online screen.

August 2021

- Consent: Created an additional consent form which stated that treatment and assessments could be either remote or in person.

January 2022

- Screen: Added additional questions regarding loss of control eating using other language to online screen to improve detection of loss of control eating.
