# Supplementary material for: Regulation of Cues vs Cognitive Behavioral Therapy for Binge Eating and Weight Loss Among Veterans: A Feasibility and Randomized Clinical Trial
Source: JAMA Netw Open. 2025 Aug 4;8(8):e2525064. doi: 10.1001/jamanetworkopen.2025.25064 (PMC12322792; doi:10.1001/jamanetworkopen.2025.25064)
Supplement: Supplement 2. — eTable 1. Differences Between ROC+BWL and CBT on Loss of Control, Binge Eating Severity, Body Mass Index, and Energy Intake eFigure. Trial Recruitment Flowchart [file jamanetwopen-e2525064-s002.pdf]

Supplemental Online Content

Boutelle KN, Afari N, Obayashi S, et al. Regulation of cues and CBT for binge eating and weight loss: a feasibility and randomized clinical trial. *JAMA Netw Open*. 2025;8(8):e2525064. doi:10.1001/jamanetworkopen.2025.25064

eTable. Differences Between ROC+BWL and CBT on Loss of Control, Binge Eating Severity, Body Mass Index, and Energy Intake
eFigure. Trial Recruitment Flowchart

This supplemental material has been provided by the authors to give readers additional information about their work.

© 2025 Boutelle KN et al. *JAMA Network Open*.

etable 1. Differences between ROC+BWL and CBT on Loss of Control, Binge Eating Severity, Body Mass Index, and Energy Intake.

	Effect of Treatment			Effect of Treatment by Time		
	**Mean (sd)**	**CI L95%, U95%**	**p**	**Mean (sd)**	**CI L95%, U95%**	**p**
**Loss Of Control**						
ROC+ vs CBT	-1.120 (0.367)	-1.870, -0.421	0.002	-1.400 (0.482)	-2.371, -0.478	0.003
Post-treatment (5-months)	-1.460 (0.282)	-2.020, -0.916	<0.001	-1.680 (0.396)	-2.474, -0.918	<0.001
6-month Follow-up (11 months)	-1.470 (0.293)	-2.050, -0.906	<0.001	-1.710 (0.416)	-2.538, -0.909	<0.001
ROC+ vs CBT at Post-treatment				0.423 (0.546)	-0.634, 1.500	0.441
ROC+ vs CBT at 6-month				0.453 (0.567)	-0.649, 1.580	0.427
**Binge Eating Severity**						
ROC+ vs CBT	-2.200 (1.253)	-4.644, 0.205	0.075	-2.650 (1.315)	-5.180, -0.044	0.047
6-month Follow-up (11 months)	1.120 (0.636)	-0.144, 2.390	0.079	0.757 (0.920)	-1.068, 2.565	0.398
ROC+ vs CBT at 6-month				0.679 (1.269)	-1.778, 3.246	0.596
**Body Mass Index**						
ROC+ vs CBT	-0.510 (0.454)	-0.992, -0.028	0.039	-0.149 (0.332)	-0.700, 0.399	0.594
Post-treatment (5-months)	-0.942 (0.097)	-1.130, -0.751	<0.001	-0.693 (0.136)	-0.960, -0.427	<0.001
6-month Follow-up (11 months)	-0.615 (0.099)	-0.809, -0.421	<0.001	-0.615 (0.139)	-0.887, -0.342	<0.001
ROC+ vs CBT at Post-treatment				-0.507 (0.193)	-0.884, -0.125	0.009
ROC+ vs CBT at 6-month				-0.010 (0.196)	-0.393 , 0.377	0.955
**Energy Intake (kCal)**						
ROC+ vs CBT	268.319 (78.445)	115.000, 422.729	0.001	270.202 (79.086)	116.000, 426.264	0.001
6-month Follow-up (11 months)	98.298 (54.514)	-6.380, 207.210	0.066	105.344 (62.531)	-15.900, 229.644	0.089
ROC+ vs CBT at 6-month				-14.727 (72.212)	-157.000, 126.554	0.837

Note: Mixed effects models included an intention to treat approach, and missing data were imputed using Joint Imputation. All models included planned covariates (age, sex, race and ethnicity, LOC episodes, and physical activity) and corresponding baseline values. Means, standard deviation (SD) and lower (2.5%) and upper (97.5%) bounds of the credible interval are presented along with the tail-probability (p).

© 2025 Boutelle KN et al. *JAMA Network Open*.

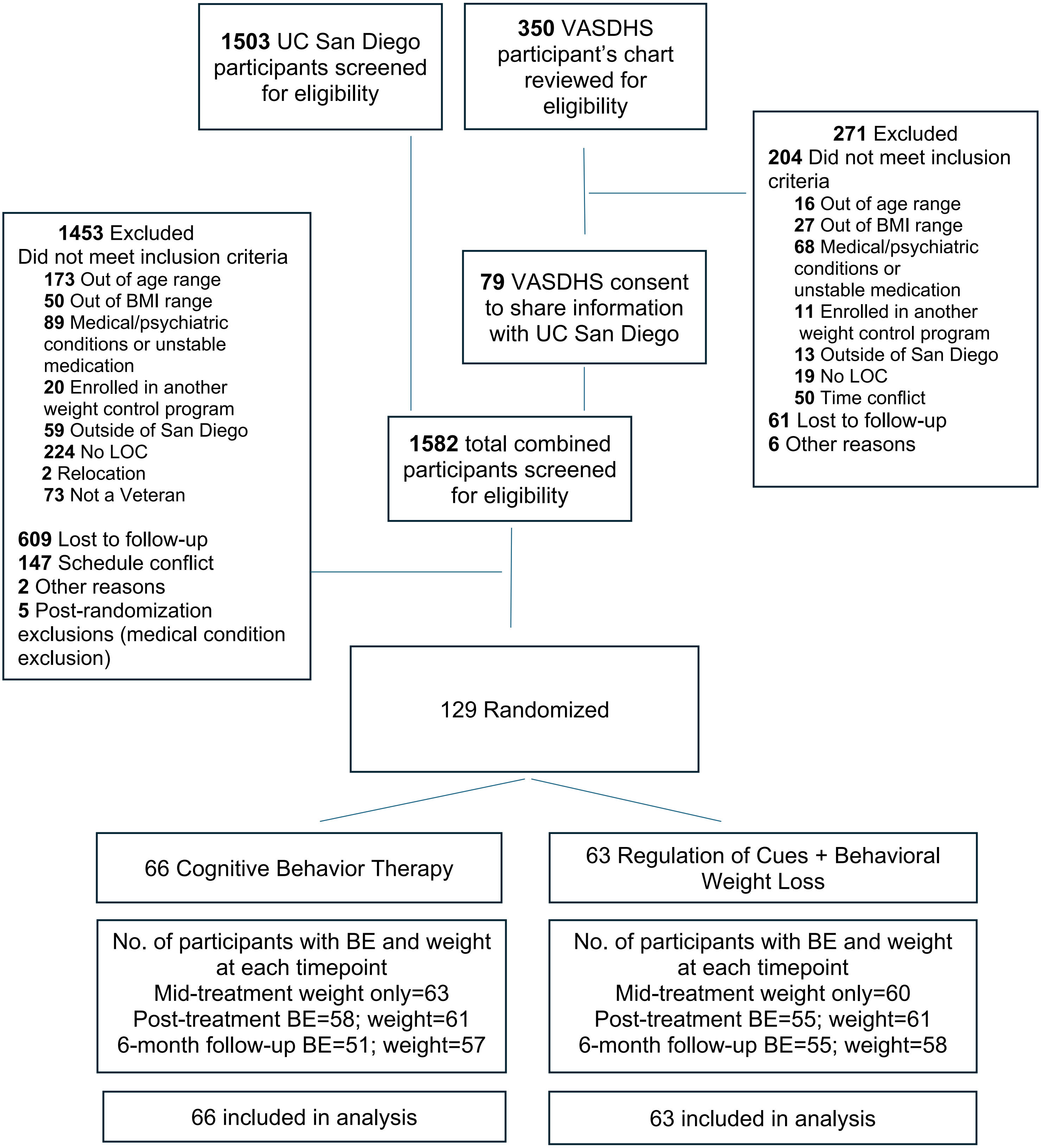

© 2025 Boutelle KN et al. *JAMA Network Open*.
