# Supplementary material for: Regulation of Cues vs Cognitive Behavioral Therapy for Binge Eating and Weight Loss Among Veterans: A Feasibility and Randomized Clinical Trial
Source: JAMA Netw Open. 2025 Aug 4;8(8):e2525064. doi: 10.1001/jamanetworkopen.2025.25064 (PMC12322792; doi:10.1001/jamanetworkopen.2025.25064)
Supplement: Supplement 3. — Data Sharing Statement [file jamanetwopen-e2525064-s003.pdf]

Data Sharing Statement

Data
Additional Information: Trial Registration: Clinicaltrials.gov NCT03678766
Data available: Yes
Data types: Deidentified participant data, Data dictionary
How to access data: Available upon request from Dr. Kerri Boutelle kboutelle@health.ucsd.edu
When available: With publication

Supporting Documents
Document types: None

Additional Information
Who can access the data: Researchers whose proposed use of the data has been approved
Types of analyses: Any purpose
Mechanisms of data availability: After approval of a proposal
